# Supplementary material for: The SensorOverlord predicts the accuracy of measurements with ratiometric biosensors
Source: Sci Rep. 2020 Oct 8;10:16843. doi: 10.1038/s41598-020-73987-0 (PMC7544824; doi:10.1038/s41598-020-73987-0)
Supplement: Supplementary file 1 [file 41598_2020_73987_MOESM1_ESM.pdf]

Supplementary information:

The SensorOverlord predicts the accuracy of measurements with ratiometric biosensors

Julian A. Stanley, Sean B. Johnsen, and Javier Apfeld*

Biology Department, Northeastern University, Boston, MA, 02115, USA

* For correspondence: j.apfeld@northeastern.edu

Supplementary Note 1

In this note we derive the map from roGFP-biosensor fluorescence-ratio (*R*) measurements into glutathione redox potential (E_{GSH}) values. We (1.1) review the general chemistry of roGFP biosensors; (1.2) derive the map between R and F_{ox}; and (1.3) derive the map between R and E_{GSH}.

(1.1) General chemistry of roGFP biosensors

roGFP biosensors include two cysteines whose thiol groups can form a reversible intramolecular disulfide bond [1]. Both reduced ($roGFP_{red}$) and oxidized ($roGFP_{ox}$) species exhibit green fluorescence but differ in the extent to which they fluoresce upon excitation at different wavelengths. Because these biosensors are derived from *Aequorea Victoria* green fluorescent protein (GFP), they exhibit two peaks in their excitation spectra, centered near 410 nm (A-band) and 470 nm (B-band), corresponding to the protonated and deprotonated forms of the biosensor's chromophore [2, 3]. The degree of chromophore protonation is influenced by the oxidation state of the two roGFP cysteines [4, 5]. As a result, the relative magnitude of these two excitation peaks differs between reduced and oxidized biosensor species. This means that when an ensemble of biosensors is excited at a given wavelength, the magnitude of their green-fluorescent emission depends not only on the number of biosensors but also on the fraction of biosensors in each oxidation state.

The fraction of oxidized biosensors (F_{ox}) provides a chemically interpretable description of the biosensor's oxidation. This fraction cannot be derived from their green-fluorescence at a single wavelength, when the biosensor concentration is not known. However, it is possible to calculate F_{ox} if one records the ratio of the biosensor's green fluorescent emission at two different wavelengths (*R*) [1, 6], as we describe in section (1.2).

The tendency of $roGFP_{ox}$ to acquire electrons and thereby become reduced into $roGFP_{red}$ is quantified by the half-cell reduction potential of the $roGFP_{red}$/$roGFP_{ox}$ couple, E_{roGFP}. This redox potential is given by the Nernst equation:

$$E_{roGFP} = E^{\circ\prime}_{roGFP} - \frac{R_{gas}T}{2F}\ln\left(\frac{[roGFP_{red}]}{[roGFP_{ox}]}\right) \quad (1)$$

where R_{gas} is the gas constant, F is the Faraday constant, T is the absolute temperature, and $E^{\circ\prime}_{roGFP}$ is the standard midpoint potential of the biosensor, which is -265 mV for roGFP1-R12 [7].

In section (1.3), we describe how one can calculate the biosensor's redox potential E_{roGFP} given F_{ox}.

The oxidation and reduction of the roGFP-family of biosensors is controlled specifically by the glutathione/glutathione disulfide redox couple, in a manner catalyzed by the enzyme glutaredoxin [1, 8, 9]. The glutathione redox couple can be thought of as a broker that mediates the indirect effects of oxidants and reductants on the thiol-disulfide balance of many proteins, including roGFPs [6]. In *C. elegans* feeding muscles, roGFP1-R12 oxidation and reduction reactions in the cytosol exhibit fast kinetics *in vivo* [6]. As a result, the glutathione and roGFP redox couples are in equilibrium; that is, the redox potential of the biosensor's couple E_{roGFP} is equal to the redox potential of the glutathione couple E_{GSH} [6]. Measuring E_{GSH} instead of R enables us to make predictions about the oxidation state of the network of cysteines trading electrons with glutathione [6].

(1.2) Derivation of the map between R and F_{ox}

(1.2.1) Dual-excitation single-emission biosensors

Assume that a fluorescent biosensor has two states, A and B, and that an individual biosensor's emission is state-dependent. Each biosensor in state A emits an intensity $i_{\lambda,A}$ and each biosensor in state B emits an intensity $i_{\lambda,B}$, where λ represents that biosensor's excitation wavelength.

If an ensemble of N_T biosensors were all in state A, the total observed intensity $I_{\lambda,A}$ would be:

$$I_{\lambda,A} = N_T \, i_{\lambda,A} \tag{2}$$

Similarly, if an ensemble of N_T biosensors were all in state B, the total observed intensity $I_{\lambda,B}$ would be:

$$I_{\lambda,B} = N_T i_{\lambda,B} \tag{3}$$

In ensembles with N_A biosensors in state A and N_B biosensors in state B, the total observed intensity I_λ will be the sum of the intensities contributed by biosensors in each state:

$$I_\lambda = \frac{N_A}{N_T} I_{\lambda,A} + \frac{N_B}{N_T} I_{\lambda,B} \tag{4}$$

Since the biosensor can only be in one of two states, $N_A + N_B = N_T$, where N_T is the total number of biosensors. Therefore:

$$I_\lambda = \frac{N_A}{N_T} I_{\lambda,A} + \left[1 - \frac{N_A}{N_T}\right] I_{\lambda,B} \tag{5}$$

We can express the probability of a biosensor being in state A as $P(A) = N_A/N_T$ and the probability of a biosensor being in state B as $P(B) = N_B/N_T = 1 - P(A)$. In a mixed population of biosensors, the total observed fluorescence intensity will be the weighted average of the intensities expected for populations of biosensors in each state:

$$I_\lambda = P(A)\, I_{\lambda,A} + [1 - P(A)]\, I_{\lambda,B} \tag{6}$$

By taking a ratiometric measurement R:

$$R = \frac{I_{\lambda_1}}{I_{\lambda_2}} = \frac{\frac{N_A}{N_T}\, I_{\lambda_1,A} + \left[1 - \frac{N_A}{N_T}\right] I_{\lambda_1,B}}{\frac{N_A}{N_T}\, I_{\lambda_2,A} + \left[1 - \frac{N_A}{N_T}\right] I_{\lambda_2,B}} \tag{7}$$

which simplifies to, in terms of $P(A)$:

$$P(A) = \frac{I_{\lambda_2} I_{\lambda_1,B} - I_{\lambda_1} I_{\lambda_2,B}}{I_{\lambda_1} I_{\lambda_2,A} - I_{\lambda_1} I_{\lambda_2,B} - I_{\lambda_2} I_{\lambda_1,A} + I_{\lambda_2} I_{\lambda_1,B}} \tag{8}$$

Now, assume that the maximum possible R measurement occurs when all biosensors are in state A (that is, when $P(A) = 1$) and that the minimum possible R occurs when all biosensors are in state B (that is, when $P(A) = 0$). We can thereby define three constants:

$$R_A = \frac{I_{\lambda_1,A}}{I_{\lambda_2,A}} \tag{9}$$

$$R_B = \frac{I_{\lambda_1,B}}{I_{\lambda_2,B}} \tag{10}$$

$$\delta_{\lambda_2} = \frac{I_{\lambda_2,A}}{I_{\lambda_2,B}} \tag{11}$$

And express $P(A)$ in terms of R and those three constants:

$$P(A) = \frac{R - R_B}{R - R_B + \delta_{\lambda_2}(R_A - R)} \tag{12}$$

Then, if biosensors in state A are oxidized, biosensors in state *B* are reduced, $\lambda_1 = 410\ nm$, and $\lambda_2 = 470\ nm$, Equation (12) becomes the map between R and F_{ox} (Equation 16):

$$R_A = R_{ox} = \frac{I_{410,ox}}{I_{470,ox}} \tag{13}$$

$$R_B = R_{red} = \frac{I_{410,red}}{I_{470,red}} \tag{14}$$

$$\delta_{\lambda_2} = \delta_{470} = \frac{I_{470,ox}}{I_{470,red}} \tag{15}$$

$$P(A) = F_{ox} = \frac{R - R_{red}}{R - R_{red} + \delta_{470}(R_{ox} - R)} \tag{16}$$

where R_{red} is the ratiometric emission of an ensemble of reduced biosensors, R_{ox} is the ratiometric emission of an ensemble of oxidized biosensors, and δ_{470} is the biosensor's dynamic range in the second wavelength, where the dynamic range $DR = \delta_{410}/\delta_{470} = R_{ox}/R_{red}$.

(1.2.2) Dual-emission single-excitation biosensors

Here, as with dual-excitation single-emission biosensors (see section 1.2.1) we assume that the fluorescent biosensor has two states, A and B, and that an individual biosensor's emission is state-dependent. In this case, however, each biosensor in state A emits an intensity $i_{\lambda,A}$ and each

biosensor in state B emits an intensity $i_{\lambda,B}$, where λ represents that biosensor's emission wavelength.

(1.2.3) Dual-excitation and dual-emission biosensors

Here, as before (see sections 1.2.1 and 1.2.2) we assume that the fluorescent biosensor has two states, A and B, and that an individual biosensor's emission is state-dependent. In this case, however, each biosensor in state A emits an intensity $i_{\lambda,A}$ and each biosensor in state B emits an intensity $i_{\lambda,B}$, where λ represents that biosensor's excitation- and emission-wavelength pair. This class of biosensors could, in principle, be built by linking two fluorescent proteins, each with well-separated maxima of fluorescence excitation and emission, with the fluorescence of the former being input-sensitive and the fluorescence of the latter being input-insensitive.

(1.3) Derivation of the map between R and E_{GSH}

The reduction potential of a two-state redox biosensor is given by the Nernst equation:

$$E_{redox\ biosensor} = E^{\circ\prime}_{redox\ biosensor} - \frac{R_{gas}T}{2F}\ln\left(\frac{[roGFP_{red}]}{[roGFP_{ox}]}\right) \tag{17}$$

In *in vivo* experiments, the concentration of the biosensor is generally not known *a priori*. The concentrations of reduced and oxidized biosensor species, however, are constrained by mass balance:

$$[roGFP] = [roGFP_{red}] + [roGFP_{ox}] \tag{18}$$

Given this constraint, we can express $E_{biosensor}$ in terms of F_{ox}:

$$[roGFP] = \frac{N_T}{Volume} = \frac{N_{red}}{Volume} + \frac{N_{ox}}{Volume} \tag{19}$$

so

$$\frac{[roGFP_{red}]}{[roGFP_{ox}]} = \frac{N_T - N_{ox}}{N_{ox}} = \frac{1 - N_{ox}/N_T}{N_{ox}/N_T} = \frac{1 - F_{ox}}{F_{ox}} \tag{20}$$

and

$$E_{redox\ biosensor} = E^{\circ'}_{redox\ biosensor} - \frac{R_{gas}T}{2F} \ln\left(\frac{1 - F_{ox}}{F_{ox}}\right) \qquad (21)$$

For roGFP biosensors, then:

$$E_{roGFP} = E^{\circ'}_{roGFP} - \frac{R_{gas}T}{2F} \ln\left(\frac{1 - F_{ox}}{F_{ox}}\right) \qquad (22)$$

For two-state ratiometric biosensors, we can use Equation (16) to write F_{ox} in terms of the fluorescence ratio R, and this expression simplifies to Equation (23):

$$E_{roGFP} = E^{\circ'}_{roGFP} - \frac{R_{gas}T}{2F} \ln\left(\delta_{\lambda_2} \frac{R_{ox} - R}{R - R_{red}}\right) \qquad (23)$$

For roGFP biosensors in equilibrium with the glutathione redox couple, then:

$$E_{GSH} = E_{roGFP} = E^{\circ'}_{roGFP} - \frac{R_{gas}T}{2F} \ln\left(\delta_{\lambda_2} \frac{R_{ox} - R}{R - R_{red}}\right) \qquad (24)$$

Supplementary Note 2

In Supplementary Note 1 we derived the map from roGFP-biosensor fluorescence-ratio (R) measurements into the fraction of oxidized biosensors (F_{ox}). Knowing F_{ox} enables calculation of the biosensor's redox potential (E_{roGFP}), which equals the glutathione redox potential (E_{GSH}) under equilibrium conditions. In this note we determine (2.1) the sensitivity of the fraction of oxidized biosensors to errors in R; and (2.2) the sensitivity of E_{GSH} values to errors in R.

(2.1) Sensitivity of the fraction of oxidized biosensors to biosensor fluorescence-ratio

For small errors in R, the sensitivity of F_{ox} to changes in R is quantified by its partial derivative with respect to R:

$$F_{ox} = \frac{R - R_{red}}{R - R_{red} + \delta_{\lambda_2}(R_{ox} - R)} \tag{1}$$

$$\frac{\partial F_{ox}}{\partial R} = \frac{\delta_{\lambda_2}(R_{ox} - R_{red})}{\left(R(\delta_{\lambda_2} - 1) - R_{ox}\delta_{\lambda_2} + R_{red}\right)^2} \tag{2}$$

where R_{red} is the ratiometric emission of an ensemble of reduced biosensors, R_{ox} is the ratiometric emission of an ensemble of oxidized biosensors, and δ_{λ_2} is the biosensor dynamic range in the second excitation band (*e.g.* in our experiments in *C. elegans*, where we excite roGFP1-R12 with bands of light $\lambda_1$and λ_2 centered at 410 nm and 470 nm, respectively, δ_{λ_2} corresponds to the dynamic range of the 470 nm excitation band [6]).

The sensitivity of F_{ox} with respect to R depends on δ_{λ_2}:

- When δ_{λ_2} is 1, the map from R to F_{ox} is linear (Figure S2-1a, black line) and, therefore, $\partial F_{ox}/\partial R$ is equal to $1/(R_{ox} - R_{red})$ throughout the range of ratio values (Figure S2-1b, black line). We note this corresponds to the special case where reduced and oxidized biosensor forms have the same fluorescence upon excitation in the second wavelength (*i.e.* λ_2 is an isosbestic point); as a result, the biosensor's fluorescence upon excitation with this wavelength does not contribute to the overall dynamic range of the biosensor fluorescence ratio (*DR*), equal to $\delta_{\lambda_1}/\delta_{\lambda_2}$.
- When δ_{λ_2} is less than 1, the map from R to F_{ox} is non-linear (Figure S2-1a, red curves). Here, the sensitivity of F_{ox} with respect to R is highest when R equals its lowest value, R_{red}, and

decreases monotonically as R approaches its maximum value, R_{ox} (Figure S2-1b, red curves). We note that in this case the biosensor's fluorescence upon excitation with each of $\lambda_1$and λ_2 contributes to the overall dynamic range of the biosensor's fluorescence ratio.

- When δ_{λ_2} is greater than 1, the map from R to F_{ox} is also non-linear (Figure S2-1a, blue curves). Here, the sensitivity of F_{ox} with respect to R is lowest when R equals its lowest value, R_{red}, and increases monotonically as R approaches its maximum value, R_{ox} (Figure S2-1a, blue curves). We note that in this case λ_1 increases the overall dynamic range of the biosensor's fluorescence ratio, but λ_2 decreases it.

(2.2) Sensitivity of the biosensor's redox potential to biosensor fluorescence-ratio

For small errors in R, the sensitivity of E_{GSH} to changes in R is quantified by its partial derivative with respect to R:

$$E_{roGFP} = E^{\circ\prime}_{roGFP} - \frac{R_{gas}T}{2F} \ln \left(\delta_{\lambda_2} \frac{R_{ox} - R}{R - R_{red}}\right) \quad (3)$$

$$\frac{\partial E_{roGFP}}{\partial R} = \frac{R_{gas}T}{2F} * \frac{R_{ox} - R_{red}}{(R_{ox} - R)(R - R_{red})} \quad (4)$$

The map from R to E_{GSH} depends on both R and δ_{λ_2} (Figure S2-1c). The sensitivity of E_{GSH} to R varies strongly with R (Figure S2-1d). We note that $\partial E_{roGFP}/\partial R$ is at its lowest value when R is halfway between its upper and lower bounds, R_{ox} and R_{red}, but increases rapidly as R approaches either one of those bounds (Figure S2-2b).

The sensitivity of E_{roGFP} to R is independent of δ_{λ_2} (Figure S2-1d). This is in contrast with the sensitivity of F_{ox} to R, which has a complex dependence on δ_{λ_2} (Figure S2-1b).

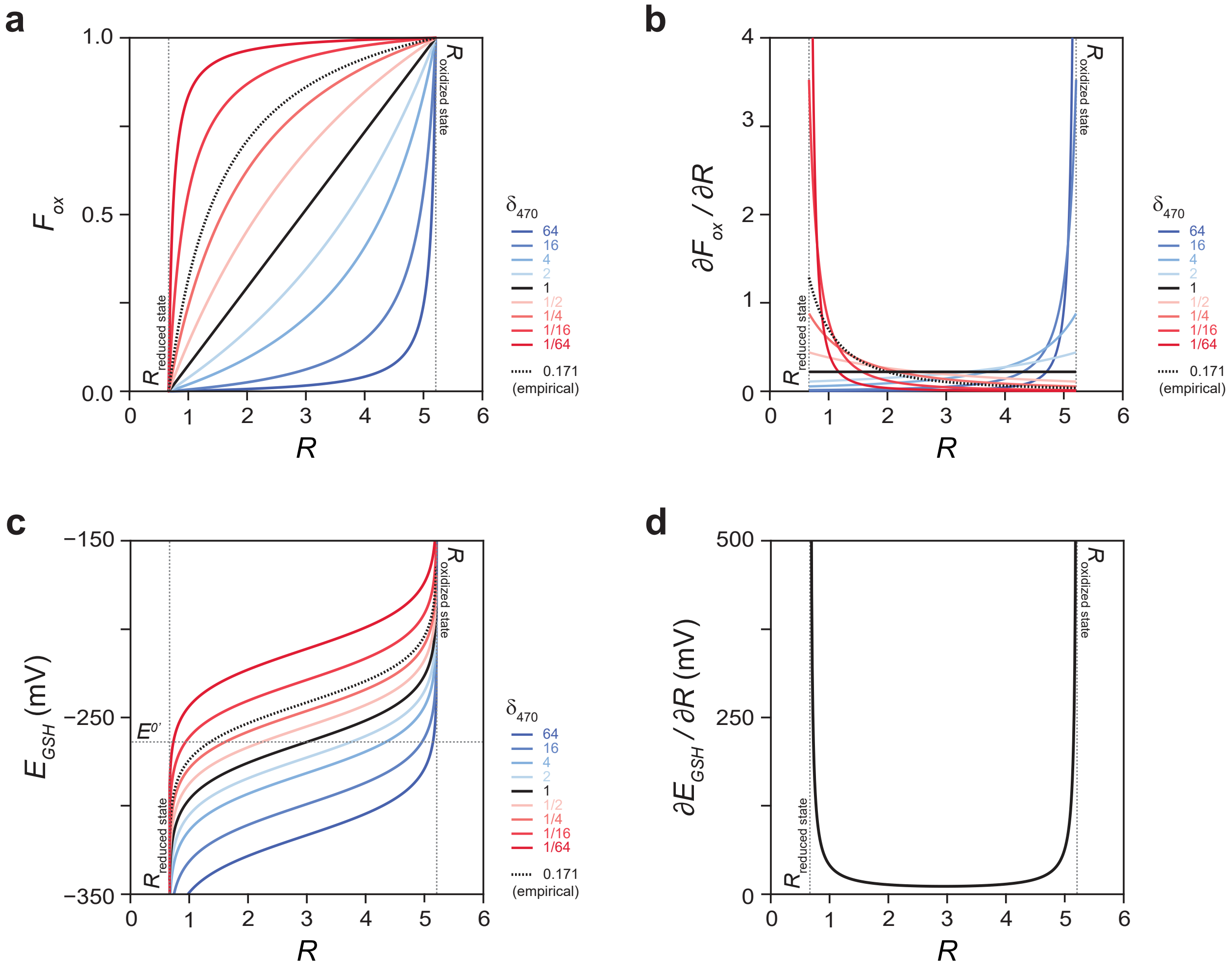

Figure S2-1: Sensitivity of the fraction of oxidized biosensors and glutathione redox potential E_{GSH} values to errors in fluorescence-ratio R

a. The conversion map from the biosensor fluorescence-ratio R to the fraction of oxidized biosensors F_{ox} depends on the ratiometric emission of the ensemble of reduced biosensors R_{red}, the ratiometric emission of the ensemble of oxidized biosensors R_{ox}, and the biosensor dynamic range in the second excitation band δ_{λ_2}.

b. The sensitivity of F_{ox} to changes in R has a complex dependence on both R and δ_{λ_2}.

c. The conversion map from R to E_{GSH} depends on both R and δ_{λ_2}.

d. The sensitivity of E_{GSH} to changes in R depends on R but is independent of δ_{λ_2}.

In panels **a**, **b***,* and **c**, the dotted curve was calculated using R_{red} R_{ox}, and δ_{λ_2}values determined empirically for our experimental setup using live *C. elegans* expressing roGFP1-R12 [6].

Supplementary Note 3

In this note, we quantify empirical errors in R in live *C. elegans* expressing the roGFP1-R12 biosensor in the cytosol of the muscles of the pharynx, the feeding organ. This retrospective analysis of thousands of fluorescence-ratio images showed that (3.1) our errors in R were relative—that is, $R_{obs} = R_{true} \times (1 + error)$—and were invariant over the range of all possible R values; and that (3.2) these relative errors vary between experiments.

(3.1) The relative error in *R* is independent of *R*

To determine whether the error in our R measurements varies with R, we quantified R errors in experiments in which the fraction of oxidized biosensors was manipulated by exposing *C. elegans* worms to different chemicals [6]. Each experiment consisted of a collection of time series where $R_{410/470}$ was measured longitudinally in individual worms first under normal conditions ("baseline") and then in response to treatment with 50 mM diamide (a thiol-specific oxidant). These animals where then either treated with 100 mM dithiothreitol (DTT, a reducing agent) or returned to normal conditions ("recovery"). Animals were placed on petri plates with modified Nematode Growth Media (to minimize background fluorescence) containing 6 mM levamisole to immobilize them.

The biosensor's green fluorescence ratio (R_{obs}) is the ratio ($R_{410/470}$) of the background-corrected observed fluorescence intensities on excitation with 410 nm and 470 nm light (I_{410} and I_{470}, respectively) captured via sequential imaging. These images of pm3 pharyngeal muscles were acquired with 4x4 binning with signal/background > 19.3 for both 410 nm and 470 nm exposures. In our images the vast majority (> 90%) of the pixels were part of the background. We therefore performed background subtraction by removing the mode intensity value of the entire image from each pixel. This procedure removed the background due to the agar and the camera noise.

Under baseline conditions R_{obs} was in a steady state or changed slowly over the measurement interval of ten minutes [6] (Figure S3-1a). Treatment with 50 mM diamide caused a new steady state in R consistent with maximal oxidation of the biosensor [6] (Figure S3-1a). Transferring animals from 50 mM diamide back to normal conditions resulted in spontaneous recovery from maximal oxidation and a new steady state in R near baseline values [6] (Figure S3-1a). To estimate R_{true}, we fitted linearly each R_{obs} time series within a 10-minute interval corresponding to the baseline or the end of each treatment. We defined the error in R as the difference between R_{obs} and R_{true} and defined the relative error in R as the error in R divided by R_{true}. The error in *R*

increased in response to diamide treatment and decreased upon recovery from diamide (Figure S3-1b). In contrast, the relative error in R was unaffected by diamide treatment or recovery (Figure S3-1c). These findings suggested the relative error in R is independent of R.

To estimate the error in R over a broader range of values, we performed the same analysis for the collection of time series in which animals were transferred first to diamide and then to DTT. We found the same pattern: diamide treatment increased the error in R and subsequent DTT treatment reduced it (Figure S3-2a); however, these treatments had only very small effects on the relative error in R (Figure S3-2b). We then re-analyzed both collections of R time series, but this time computed the errors in the observed $R_{470/410}$ values using the same approach as for the errors in $R_{410/470}$. Because $R_{470/410}$ is the inverse of $R_{410/470}$, this approach enabled us to eliminate many gaps in R coverage. The observed fluorescence ratios and their inverses varied over a wide range of values and exhibited large difference in their absolute errors (Figure S3-2a,c); however, their relative errors exhibited only very small differences (Figure S3-2b,d). These results confirm that in our experiments, the relative error in R was independent of R.

(3.2) The relative error in *R* can vary between experiments

To determine whether the relative error in R varies between experiments, we followed the same approach as in (3.1) and analyzed three additional collections of time series of animals under baseline conditions. We found that the relative error in R varied between the baselines of the five experiments by up to three-fold (Figure S3-3). Differences in the proportion of animals moving during imaging account for most of the variation in the relative error in *R* across experiments (S.B.J, J.A.S, and J.A., manuscript in preparation).

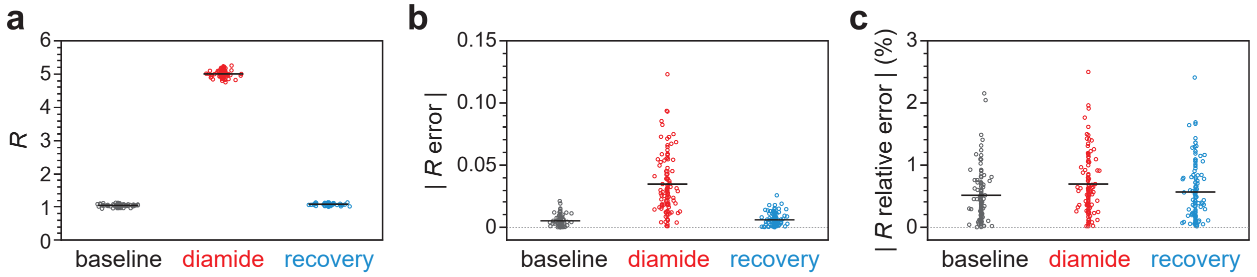

Figure S3-1: The error in R is relative

a. $R_{410/470}$ measurements in *C. elegans* worms expressing roGFP1-R12 in feeding muscles under normal conditions (“baseline”), in response to treatment with 50 mM diamide, and upon return to normal conditions (“recovery”).

b. The absolute value of the error in R varies between baseline, diamide treatment, and recovery ($P < 0.0001$, ANOVA).

c. The absolute value of the relative error in R does not vary between baseline, diamide treatment, and recovery conditions ($P > 0.05$, ANOVA).

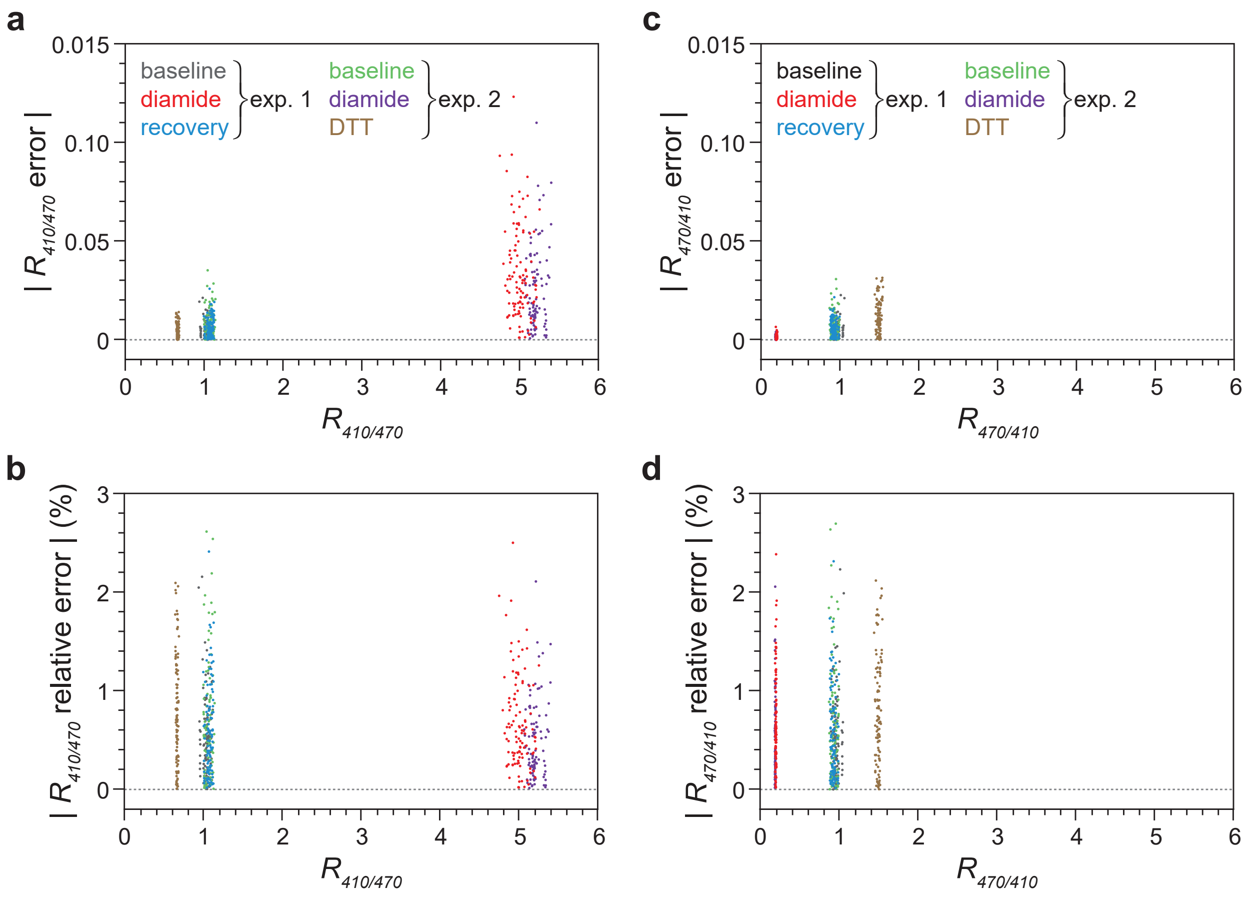

Figure S3-2: The relative error in *R* is independent of *R* over a broad range of *R* values

a. The absolute value of the error in $R_{410/470}$ varies with $R_{410/470}$ (*P* < 0.0001, linear regression).

b. The absolute value of the relative error in $R_{410/470}$ does not vary with $R_{410/470}$ (*P* > 0.05, linear regression).

c. The absolute value of the error in $R_{470/410}$ varies with $R_{470/410}$ (*P* < 0.0001, linear regression).

d. The absolute value of the relative error in $R_{470/410}$ does not vary with $R_{470/410}$ (*P* > 0.05, linear regression).

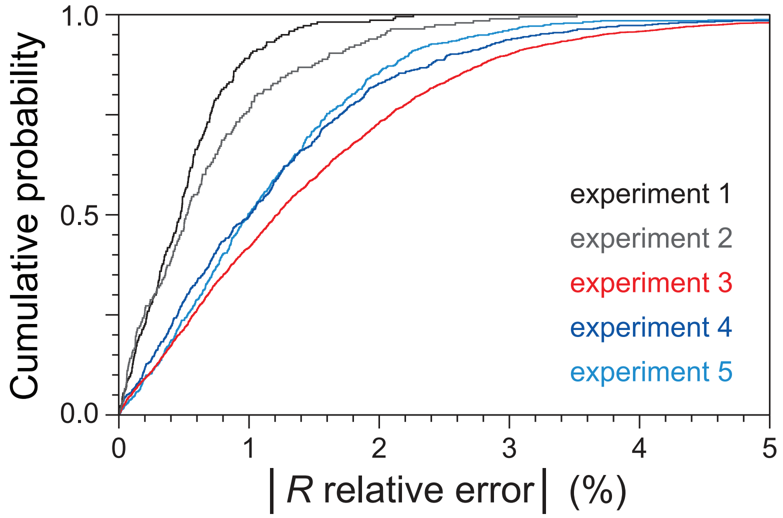

Figure S3-3: The relative error in R can vary between experiments

Cumulative distributions of the absolute value of the relative error in $R_{410/470}$ in five experiments consisting of baseline measurements of $R_{410/470}$. The absolute value of the relative error in $R_{410/470}$ varied between pairs of experiments (*P* < 0.0001, Turkey HSD test), except for experiments 1 and 2 and experiments 4 and 5, which did not exhibit differences (*P* > 0.05, Turkey HSD test).

Supplementary Note 4

In this note, we build on the framework described in Supplementary Note 1 to model how the biochemical and biophysical properties of roGFP biosensors affect the relationship between the empirical precision of our fluorescence-ratio measurements and the accuracy of individual E_{GSH} observations: we (4.1) present the overall approach; (4.2) show how E_{GSH} measurement inaccuracy is sensitive to the biochemical and biophysical variables that map R to E_{GSH}; and (4.3) extend this analysis to other types of ratiometric two-state biosensors.

(4.1) Overall approach

In Supplementary Note 1.3 we derived the function mapping the ratiometric emission (R) of dual-excitation single emission roGFP biosensors and the reduction potential of the glutathione-glutathione disulfide couple E_{GSH}:

$$E_{GSH} = E_{roGFP} = E^{\circ\prime}_{roGFP} - \frac{R_{gas}T}{2F}\ln\left(\delta_{\lambda_2}\frac{R_{ox} - R}{R - R_{red}}\right) \qquad (1)$$

The function mapping R to E_{GSH} depends on physical, biochemical, and biophysical parameters. These include (a) physical constants and variables: the gas constant R_{gas}, the Faraday constant F, and the absolute temperature T; (b) biochemical variables: the standard midpoint potential of the biosensor $E^{\circ\prime}_{roGFP}$; and (c) biophysical variables: the ratiometric emission of an ensemble of reduced biosensors R_{red}, the ratiometric emission of an ensemble of oxidized biosensors R_{ox}, and the biosensor's dynamic range in the second excitation wavelength δ_{λ_2}.

In the main text, we modeled how the empirical error in R measurement influences the accuracy of individual E_{GSH} measurements (Figure 1g). In this note, we model how that relationship is influenced by each of the biochemical and biophysical variables mapping R to E_{GSH}.

(4.2) Sensitivity of E_{GSH} measurement inaccuracy to the biochemical and biophysical variables that map R to E_{GSH}

In this analysis, we changed the values of individual parameters of the function mapping R to E_{GSH} while holding other parameters constant. We then determined how the inaccuracy of our E_{GSH} measurements would be affected by a 2.8%-fold error in R. This relative error corresponds to the 0.025 and 0.975 quartiles of our empirical distribution of relative errors in R (the median relative error in R was zero) (Figure 1f). Thus, the E_{GSH} inaccuracy we calculated is the maximum absolute

difference between E_{True} and either the upper or lower bounds of the interval encompassing 95% of the $E_{Observed}$ values.

As a starting point we considered a hypothetical roGFP biosensor with $E°'$ = -250 mV. Other parameters were set as follows: R_{red} = 1, R_{ox} = 5, and δ_{λ_2} = 1. We note the constraints between certain parameters given by the biosensor's dynamic range DR:

$$DR = \frac{R_{ox}}{R_{red}} = \frac{\delta_{\lambda_1}}{\delta_{\lambda_2}} \quad (2)$$

Changing the biosensor's midpoint potential shifts the x-axis of the map between the true value of E_{GSH} and E_{GSH} inaccuracy (Figure S4-1a). The map between $E_{GSH} - E°'$ and E_{GSH} inaccuracy is unaffected by the value of $E°'$.

Changing the biosensor's δ_{λ_2} while holding DR constant also shifted the x-axis of the map between the true value of E_{GSH} and E_{GSH} inaccuracy (Figure S4-1b). An x-fold change in δ_{λ_2} while holding DR constant shifts the x-axis of that map by $-\frac{R_{gas}T}{2F}\ln x$.

Changing the biosensor's δ_{λ_1} without the constraint of holding DR constant had a more complex effect (Figure S4-1c). Increasing δ_{λ_1} (and thereby DR) always lowered E_{GSH} inaccuracy. In addition, it decreased the E_{GSH} value that maps to the lowest E_{GSH} inaccuracy. Changing δ_{λ_2} without the constraint of holding DR constant had a similar effect to changing δ_{λ_1} (Figure S4-1d). At a given DR value, the curves mapping E_{GSH} to E_{GSH} inaccuracy are identical if one changed either δ_{λ_1} or δ_{λ_2} except for a shift in the x-axis (due to changing δ_{λ_2}at constant DR, described above).

In summary, our sensitivity analysis indicates that the optimal strategy for minimizing E_{GSH} inaccuracy with a given biosensor and error level in R is to select excitation wavelengths that maximize the biosensor's dynamic range DR. Choosing a combination of wavelengths that includes a wavelength at the isosbestic point of a biosensor (*i.e.* where δ = 1) provides a linear map between R and the biosensor's fraction oxidized F_{ox} (Figure S2-1a); however, such wavelength combinations would always result in higher E_{GSH} inaccuracy than combinations with a higher DR.

(4.3) Generalization to other types of ratiometric biosensors

In Supplementary Note 1.2, we showed that the function mapping R to E_{GSH} (equation 1 in this note) generalizes to dual-emission single-excitation biosensors, and to dual-excitation and dual-emission biosensors; and in Supplementary Note 7 we show that a function of the same general form applies to pH and ligand-binding biosensors. Therefore, the conclusions of the sensitivity analysis presented in this Supplementary Note apply to all these types of biosensors.

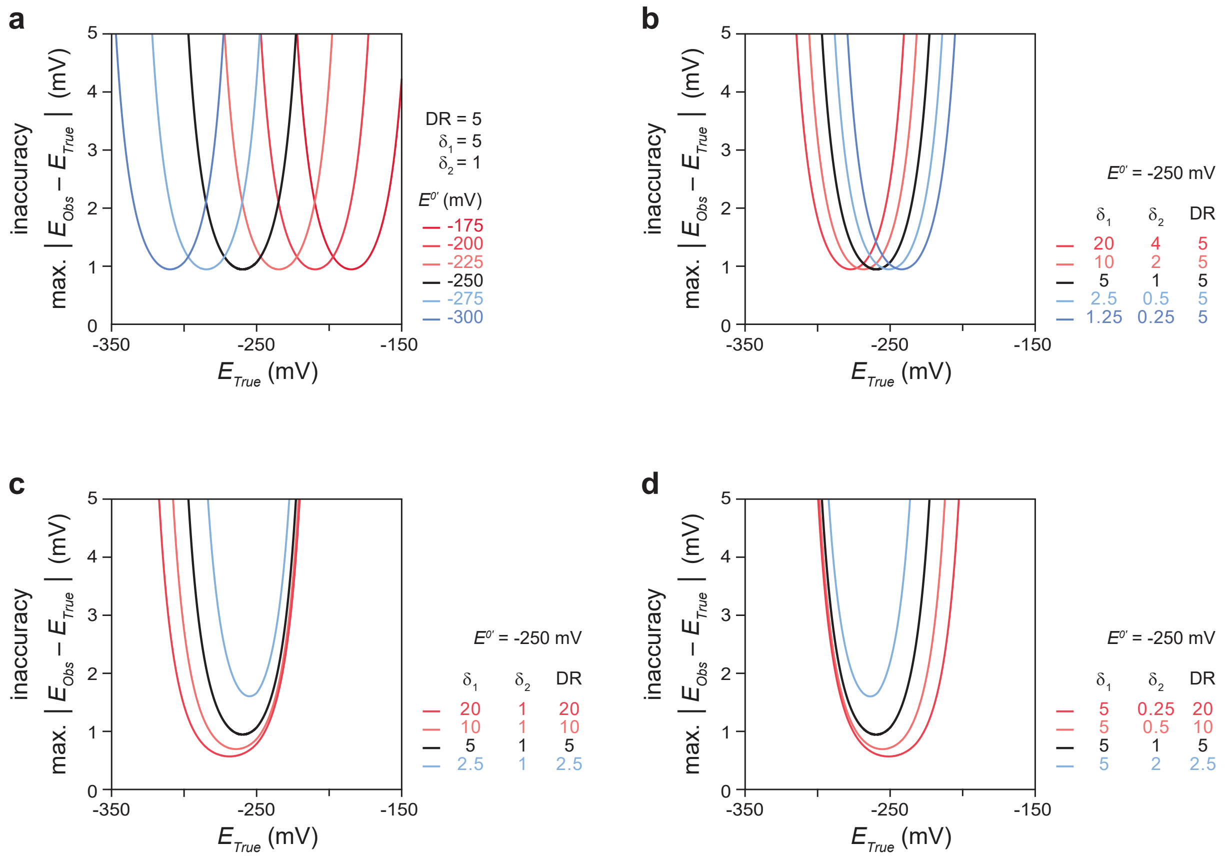

Figure S4-1: Sensitivity of E_{GSH} measurement inaccuracy to the biochemical and biophysical variables that map R to E_{GSH}

a. Sensitivity of E_{GSH} measurement inaccuracy (the maximum absolute difference between E_{True} and $E_{Observed}$) to the standard midpoint potential of the biosensor $E°'$, when other parameters mapping R to E_{GSH} are held constant.

b. Sensitivity of E_{GSH} measurement inaccuracy to the biosensor's dynamic range in the first and second excitation wavelengths, δ_1 and δ_2, respectively, when holding constant the biosensor's dynamic range DR and the biosensor's $E°'$.

c. Sensitivity of E_{GSH} measurement inaccuracy to the biosensor's dynamic range in the first excitation wavelength δ_1, when holding constant the biosensor's dynamic range in the second excitation wavelength δ_2 and the biosensor's $E°'$.

d. Sensitivity of E_{GSH} measurement inaccuracy to the biosensor's dynamic range in the second excitation wavelength δ_2, when holding constant the biosensor's dynamic range in the first excitation wavelength δ_1 and the biosensor's $E°'$.

In all panels, the relative error in R was set to 2.8%-fold, which corresponds to the 0.025 and 0.975 quartiles of the empirical distribution of relative errors in R in our experiments with roGFP1-R12 in the feeding muscles of *C. elegans* (Figure 1f).

Supplementary Note 5

In Supplementary Note 1 we derived the function mapping the ratiometric emission (R) of dual-excitation single emission roGFP biosensors and the reduction potential of the glutathione-glutathione disulfide couple E_{GSH}. That map depends on physical, biochemical, and biophysical parameters, which either quantify intrinsic properties of the biosensor or vary between microscope setups. Knowledge of these parameters enables modeling how the precision of R measurements with a biosensor determines the range of E_{GSH} values that is possible to measure with that biosensor at a given inaccuracy level. In this note, we (5.1) determine the minimal set of parameters and constraints between parameters needed to predict the influence of relative errors in R on E_{GSH} measurement error; (5.2) estimate the values of those parameters for eleven roGFP-based biosensors with known midpoint potentials and fluorescence spectra; and (5.3) determine the E_{GSH} inaccuracy we would expect to observe had we measured E_{GSH} in the feeding muscles of live *C. elegans* with each of those biosensors instead of roGFP-R12.

(5.1) Which of the parameters mapping a biosensor's fluorescence ratio R to E_{GSH} are needed to model how relative errors in R influence E_{GSH} measurement error?

<u>(5.1.1) What are the values of the parameters mapping a biosensor's fluorescence ratio R to E_{GSH}?</u>

The function mapping the ratiometric emission (R) of dual-excitation single emission roGFP biosensors and the reduction potential of the glutathione-glutathione disulfide couple E_{GSH}, was derived in Supplementary Note 1.3:

$$E_{GSH} = E_{roGFP} = E^{\circ\prime}_{roGFP} - \frac{R_{gas}T}{2F}\ln\left(\delta_{\lambda_2}\frac{R_{ox} - R}{R - R_{red}}\right) \qquad (1)$$

That map depends on physical, biochemical, and biophysical parameters. The physical parameters are generally known; they include the gas constant R_{gas}, the Faraday constant F, and the absolute temperature T. The biochemical parameter, the standard midpoint potential of the biosensor $E^{\circ\prime}_{roGFP}$, is often known from published reports. The biophysical parameters include the biosensor's dynamic range in the second excitation wavelength δ_{λ_2}, the ratiometric emission of an ensemble of reduced biosensors R_{red}, and the ratiometric emission of an ensemble of oxidized biosensors R_{ox}. These three parameters can be determined empirically, as we have done previously in live *C. elegans* expressing roGFP1-R12 [6], but in most cases they are not known. The value of δ_{λ_2} can be determined from the fluorescence spectra of reduced and oxidized biosensors (section 5.1.2). These spectra also produce R_{ox} and R_{red} values (section 5.1.2). However, these

values vary from one imaging set up to another. Because R_{ox} and R_{red} values are instrument specific, the biosensor's ratiometric emission R collected in a microscope cannot be mapped to E_{GSH} using the R_{ox} and R_{red} values obtained from a different instrument, such as spectrofluorometer used to collect the excitation spectra of reduced and oxidized biosensor species. The variation of R_{ox} and R_{red} across instruments, however, is constrained by the biophysical properties of the biosensor at the excitation wavelengths λ_1 and λ_2, such that the ratio of R_{ox} to R_{red} (the biosensor's overall dynamic range, DR) is constant. DR can be determined from the fluorescence spectra of reduced and oxidized biosensors (section 5.1.2). Therefore, in order to map R to E_{GSH} for a given biosensor, either R_{ox} or R_{red} must be determined empirically in the same instrument where that biosensor's R is measured.

Sometimes, all parameters mapping a biosensor's ratiometric emission in a given instrument to E_{GSH} values are known, except for R_{ox} and R_{red}. Even though in those cases it is not possible to map a biosensor's R to E_{GSH}, it is still desirable to determine how relative errors in that biosensor's R would influence E_{GSH} measurement error. For example, such analysis may enable comparison of the expected E_{GSH} inaccuracy of different biosensors under otherwise identical microscopy conditions. In section (5.1.3), we demonstrate that knowledge of the biosensor's overall dynamic range DR is sufficient to model how relative errors in a biosensor's R influence E_{GSH} measurement error with that biosensor when the values of R_{red} and R_{ox} are not known.

(5.1.2) Obtaining δ_{λ_2} and R_{ox}/R_{red} from the excitation spectra of reduced and oxidized biosensor species

Equation (1) can be reparametrized in terms of the biosensor's overall dynamic range DR:

$$DR = \frac{R_{ox}}{R_{red}} = \frac{\delta_{\lambda_1}}{\delta_{\lambda_2}} \quad (2)$$

$$E_{GSH} = E_{roGFP} = E^{\circ\prime}_{roGFP} - \frac{R_{gas}T}{2F} \ln\left(\delta_{\lambda_2} \frac{DR - R/R_{red}}{R/R_{red} - 1}\right) \quad (3)$$

where δ_{λ} is the ratio of the biosensor's emission when excited at λ in the oxidized and reduced states:

$$\delta_{\lambda} = \frac{i_{\lambda,ox}}{i_{\lambda,red}} \quad (4)$$

δ_λ is, therefore, independent of the illumination power at λ and should be invariant across instruments.

The overall dynamic range DR of a dual-excitation biosensor is the ratio of the $\delta_\lambda$values at each excitation wavelength. DR is also independent of the illumination power at λ_1 and λ_2, and should be invariant across instruments.

(5.1.3) Modeling how relative errors in R influence E_{GSH} measurement error when the values of R_{red} and R_{ox} are not known but their ratio is known

Unlike DR and δ_λ, R_{ox} and R_{red} are instrument-dependent and must be determined empirically for each imaging instrument. We note that since $DR = R_{ox}/R_{red}$, then R_{ox} can be determined from R_{red} and vice-versa. In this section, we show that knowledge of DR is sufficient to model how relative errors in R influence E_{GSH} measurement error when all parameters mapping the biosensor's ratiometric emission to E_{GSH} are known except for R_{red} and R_{ox}.

To understand the origin of the instrument-dependence of R_{ox} and R_{red} let us consider two imaging microscopes, M1 and M2, with identical excitation light intensity at λ_1, but with M2 having only a fraction f as much excitation light intensity at λ_2 as M1. Under these conditions, a sample will exhibit the same biosensor fluorescence intensity at λ_1 in M1 and M2:

$$I_{\lambda_1,M2} = I_{\lambda_1,M1} \tag{5}$$

However, that sample will exhibit a fraction f of the fluorescence intensity at λ_2 in M1 relative to M2.

$$I_{\lambda_2,M2} = f \times I_{\lambda_2,M1} \tag{6}$$

Thus, the sample's fluorescence ratio will differ across imaging microscopes by the illumination factor f:

$$R_{M1} = \frac{I_{\lambda_1,M1}}{I_{\lambda_2,M1}} \tag{7}$$

$$R_{M2} = \frac{I_{\lambda_1,M2}}{I_{\lambda_2,M2}} \quad (8)$$

Thus,

$$R_{M1} = f \times R_{M2} \quad (9)$$

As a result of differences in illumination across instruments, R_{ox} and R_{red} will be affected similarly by the illumination factor f. That is, under different imaging conditions:

$$R_{ox}' = f \times R_{ox} \quad (10)$$

$$R_{red}' = f \times R_{red} \quad (11)$$

and

$$R' = f \times R \quad (12)$$

In Supplementary Note 3 we showed that, in our experiments, the relative error in R was independent of R. That is:

$$R_{observed} = R_{true} \times (1 + error) \quad (13)$$

Thus $error$ is unaffected by the illumination factor f:

$$R'_{observed} = f \times R_{observed} = f \times R_{true} \times (1 + error) = R_{true}' \times (1 + error) \quad (14)$$

Because the relative error in R is equal to the relative error in R', its effect on the error in E_{GSH} is the same when applied to the function mapping R to E_{GSH} (equation 16) as it is when applied to the function mapping R' to E_{GSH} (equation 20), as shown below:

$$\Delta E_{GSH} = E_{observed} - E_{true} \tag{15}$$

Substituting equation (3),

$$\Delta E_{GSH} = \left[E^{\circ\prime}_{roGFP} - \frac{R_{gas}T}{2F}\ln\left(\delta_{\lambda_2}\frac{DR - \frac{R_{observed}{}'}{R_{red}{}'}}{\frac{R_{observed}{}'}{R_{red}{}'} - 1}\right)\right] - \left[E^{\circ\prime}_{roGFP} - \frac{R_{gas}T}{2F}\ln\left(\delta_{\lambda_2}\frac{DR - \frac{R_{true}{}'}{R_{red}{}'}}{\frac{R_{true}{}'}{R_{red}{}'} - 1}\right)\right] \tag{16}$$

Substituting equation (14),

$$\Delta E_{GSH} = \left[E^{\circ\prime}_{roGFP} - \frac{R_{gas}T}{2F}\ln\left(\delta_{\lambda_2}\frac{DR - \frac{R_{true}{}' \times (1 + error)}{R_{red}{}'}}{\frac{R_{true}{}' \times (1 + error)}{R_{red}{}'} - 1}\right)\right] - \left[E^{\circ\prime}_{roGFP} - \frac{R_{gas}T}{2F}\ln\left(\delta_{\lambda_2}\frac{DR - \frac{R_{true}{}'}{R_{red}{}'}}{\frac{R_{true}{}'}{R_{red}{}'} - 1}\right)\right] \tag{17}$$

Substituting with equations (11) and (14), we obtain:

$$\Delta E_{GSH} = \left[E^{\circ\prime}_{roGFP} - \frac{R_{gas}T}{2F}\ln\left(\delta_{\lambda_2}\frac{DR - \frac{f \times R_{true} \times (1 + error)}{f \times R_{red}}}{\frac{f \times R_{true} \times (1 + error)}{f \times R_{red}} - 1}\right)\right] - \left[E^{\circ\prime}_{roGFP} - \frac{R_{gas}T}{2F}\ln\left(\delta_{\lambda_2}\frac{DR - \frac{f \times R_{true}}{f \times R_{red}}}{\frac{f \times R_{true}}{f \times R_{red}} - 1}\right)\right] \tag{18}$$

$$\Delta E_{GSH} = \left[E^{\circ\prime}_{roGFP} - \frac{R_{gas}T}{2F}\ln\left(\delta_{\lambda_2}\frac{DR - \frac{R_{true} \times (1 + error)}{R_{red}}}{\frac{R_{true} \times (1 + error)}{R_{red}} - 1}\right)\right] - \left[E^{\circ\prime}_{roGFP} - \frac{R_{gas}T}{2F}\ln\left(\delta_{\lambda_2}\frac{DR - \frac{R_{true}}{R_{red}}}{\frac{R_{true}}{R_{red}} - 1}\right)\right] \tag{19}$$

Substituting equation (13),

$$\Delta E_{GSH} = \left[E^{\circ\prime}_{roGFP} - \frac{R_{gas}T}{2F}\ln\left(\delta_{\lambda_2}\frac{DR - \frac{R_{observed}}{R_{red}}}{\frac{R_{observed}}{R_{red}} - 1}\right)\right] - \left[E^{\circ\prime}_{roGFP} - \frac{R_{gas}T}{2F}\ln\left(\delta_{\lambda_2}\frac{DR - \frac{R_{true}}{R_{red}}}{\frac{R_{true}}{R_{red}} - 1}\right)\right] \tag{20}$$

$$\Delta E_{GSH} = E_{observed} - E_{true} \tag{21}$$

We conclude that knowledge of DR is sufficient to model how relative errors in R influence E_{GSH} measurement error, when all parameters mapping the biosensor's ratiometric emission to E_{GSH} are known except for R_{red} and R_{ox}. We can therefore model how the relative errors in R

would influence the expected E_{GSH} inaccuracy of different biosensors under otherwise identical microscopy conditions.

(5.2) Obtaining $\boldsymbol{\delta_{\lambda_1}}$, $\boldsymbol{\delta_{\lambda_2}}$, $\boldsymbol{R_{ox}}$, $\boldsymbol{R_{red}}$, and $\boldsymbol{DR}$ parameter values for eleven roGFP-based biosensors with known midpoint potentials and fluorescence spectra

For twenty-four roGFP biosensors, we attempted to obtain the fluorescence excitation spectra of reduced and oxidized biosensor species and the biosensor $E^{\circ\prime}$ value from published data, and from personal correspondence with authors when the spectra had not been published (Table S5-1). While $E^{\circ\prime}$ values for all of these biosensors have been reported; the spectra of reduced and oxidized biosensor species of only eleven of these biosensors have been collected, precluding further analysis of the remaining biosensors. Spectra were digitized with WebPlotDigitizer software [10]. roGFP biosensors exhibit two peaks in their excitation spectra, centered near 410 nm (A-band) and 470 nm (B-band) (Figure S5-1, first column). Two sets of δ_{λ_1}, δ_{λ_2}, R_{ox}, R_{red}, and DR parameter values were collected from the intensity spectra of reduced and oxidized species of each roGFP biosensor. The first set matches the bandwidths of the excitation filters in our microscopy setup, and the latter matches the wavelengths of widely used lasers. For the former, the signal at λ_1 and λ_2 were the average signals when excited over the intervals 410±15 nm and 470±10 nm, respectively. For the latter, the biosensors signal at λ_1 and λ_2 were the average signals when excited over the intervals 405±1 nm and 488±1 nm, respectively. The parameters of each biosensor were determined as described in (5.1.2). These parameters varied significantly between biosensors (Table S5-2 and Figure S5-1, second column).

(5.3) Predicted $\boldsymbol{E_{GSH}}$ inaccuracy of eleven roGFP-based biosensors

Using the δ_{λ_2}, R_{ox}, and R_{red} parameters obtained in (5.2), we mapped R to E_{GSH} for eleven roGFP-based biosensors with known midpoint potentials and fluorescence spectra. We applied the SensorOverlord framework to determine the E_{GSH} inaccuracy that we would expect to observe at each E_{GSH} value with each of those biosensors, given our empirical distribution of relative errors in R when measuring E_{GSH} in the feeding muscles of live *C. elegans* expressing roGFP-R12 (Figure S5-1, third column).

Table S5-1: roGFP biosensors

Biosensor		$E^{0'}$ (mV)		Spectra?	
roGFP1	[5]	-287	[5, 11]	Yes	[5, 12]
roGFP2	[5]	-272	[5]	Yes	[1, 5, 13-16]
roGFP3	[5]	-299	[5]	Yes	[13]
roGFP4	[5]	-286	[5]	Yes	[13]
roGFP5	[5]	-296	[5]	Yes	[13]
roGFP6	[5]	-280	[5]	Yes	[13]
roGFP1-R1	[7]	-269	[7]	No	
roGFP1-R3	[7]	-282	[7]	No	
roGFP1-R7	[7]	-268	[7]	No	
roGFP1-R8	[7]	-284	[7]	No	
roGFP1-R9	[7]	-278	[7]	Yes	[17]
roGFP1-R10	[7]	-284	[7]	No	
roGFP1-R11	[7]	-275	[7]	No	
roGFP1-R12	[7]	-265	[7]	Yes	James Remington (personal communication)
roGFP1-R14	[7]	-263	[7]	No	
roGFP1-iL	[11]	-229 +/- 5	[11]	Yes	[11]
roGFP1-iE	[11]	-236 +/- 7	[11]	Yes	[11]
roGFP1-iQ	[11]	-239 +/- 6	[11]	No	
roGFP1-iH	[11]	-238 +/- 4	[11]	No	
roGFP1-iR	[11]	-237 +/- 5	[11]	No	
roGFP1-iS	[11]	-240 +/- 3	[11]	No	
roGFP1-iD	[11]	-246 +/- 1	[11]	No	
roGFP2-iL	[14]	-238	[14]	Yes	[14]
grx1-roGFP2	[18]	-280	[18]	No	

Table S5-2: Biosensor parameter values used for mapping R to E_{GSH}

Biosensor	$E^{\circ\prime}$ (mV)	Parameter set 1 λ_1 = 410 ± 15 nm and λ_2 = 470 ± 10 nm					Parameter set 2 λ_1 = 405 ± 1 nm and λ_2 = 488 ±1 nm					Spectra source
		DR	δ_{410}	δ_{470}	R_{red}	R_{ox}	DR	δ_{405}	δ_{488}	R_{red}	R_{ox}	
roGFP1	-287	6.08	1.64	0.27	3.67	22.33	3.96	1.66	0.42	5.25	20.77	[4]
roGFP1	-287	6.67	1.67	0.25	3.67	24.49	4.72	1.65	0.35	4.98	23.49	[5]
roGFP1	-287	4.97	1.24	0.25	1.42	7.06	3.99	1.36	0.34	2.18	8.69	[12]
roGFP1-iE	-236	3.12	1.47	0.47	1.02	3.18	1.68	1.65	0.98	3.24	5.45	[11]
roGFP1-iL	-229	4.13	1.36	0.33	2.15	8.87	2.89	1.39	0.48	4.09	11.81	[11]
roGFP1-R12 *	-265	7.81	1.33	0.171	0.667	5.207						None
roGFP1-R12	-265	5.01	1.30	0.26	1.42	7.12	4.04	1.37	0.34	2.21	8.93	James Remington (personal communication)
roGFP1-R9	-278	4.92	1.28	0.26	1.44	7.09	4.08	1.34	0.33	2.26	9.21	[17]
roGFP2	-272	6.33	2.22	0.35	0.15	0.95	7.89	3.08	0.39	0.09	0.71	[5]
roGFP2-iL	-238	2.50	1.63	0.65	0.18	0.45	2.69	2.02	0.75	0.13	0.35	[14]
roGFP3	-299	4.25	0.98	0.23	0.97	4.12	2.95	1.00	0.34	1.37	4.04	[13]
roGFP4	-286	2.11	0.74	0.35	0.09	0.19	2.40	0.91	0.38	0.05	0.12	[13]
roGFP5	-296	6.92	1.11	0.16	1.04	7.20	4.27	1.15	0.27	1.87	7.98	[13]
roGFP6	-280	3.33	1.20	0.36	0.12	0.40	3.67	1.43	0.39	0.09	0.33	[13]

* δ_{λ_1}, δ_{λ_2}, R_{ox}, R_{red}, and DR values were determined empirically in *C. elegans* expressing roGFP1-R12 in the feeding muscles [6].

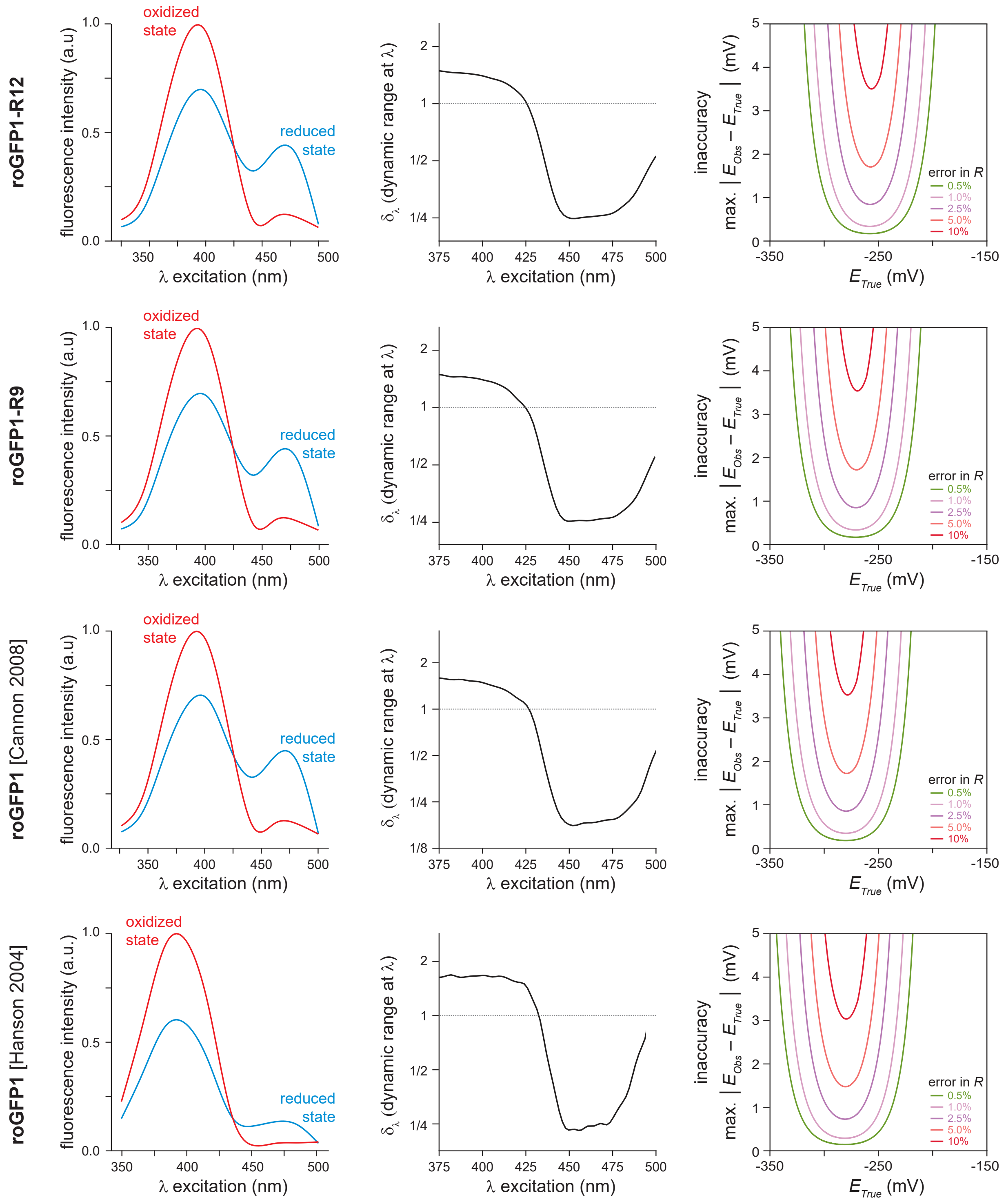

Figure S5-1: Predicted E_{GSH} measurement inaccuracy of eleven roGFP-based biosensors

In this analysis for eleven roGFP biosensors, we show in each row a plot of a roGFP biosensor's emission when excited at a wavelength λ in the oxidized and reduced states (column 1); a plot of the ratio of those emissions, the biosensor's dynamic range δ_λ, at each wavelength λ (column 2); and a plot of the predicted E_{GSH} measurement inaccuracy with that biosensor at each wavelength λ for different relative error in R values (column 3).

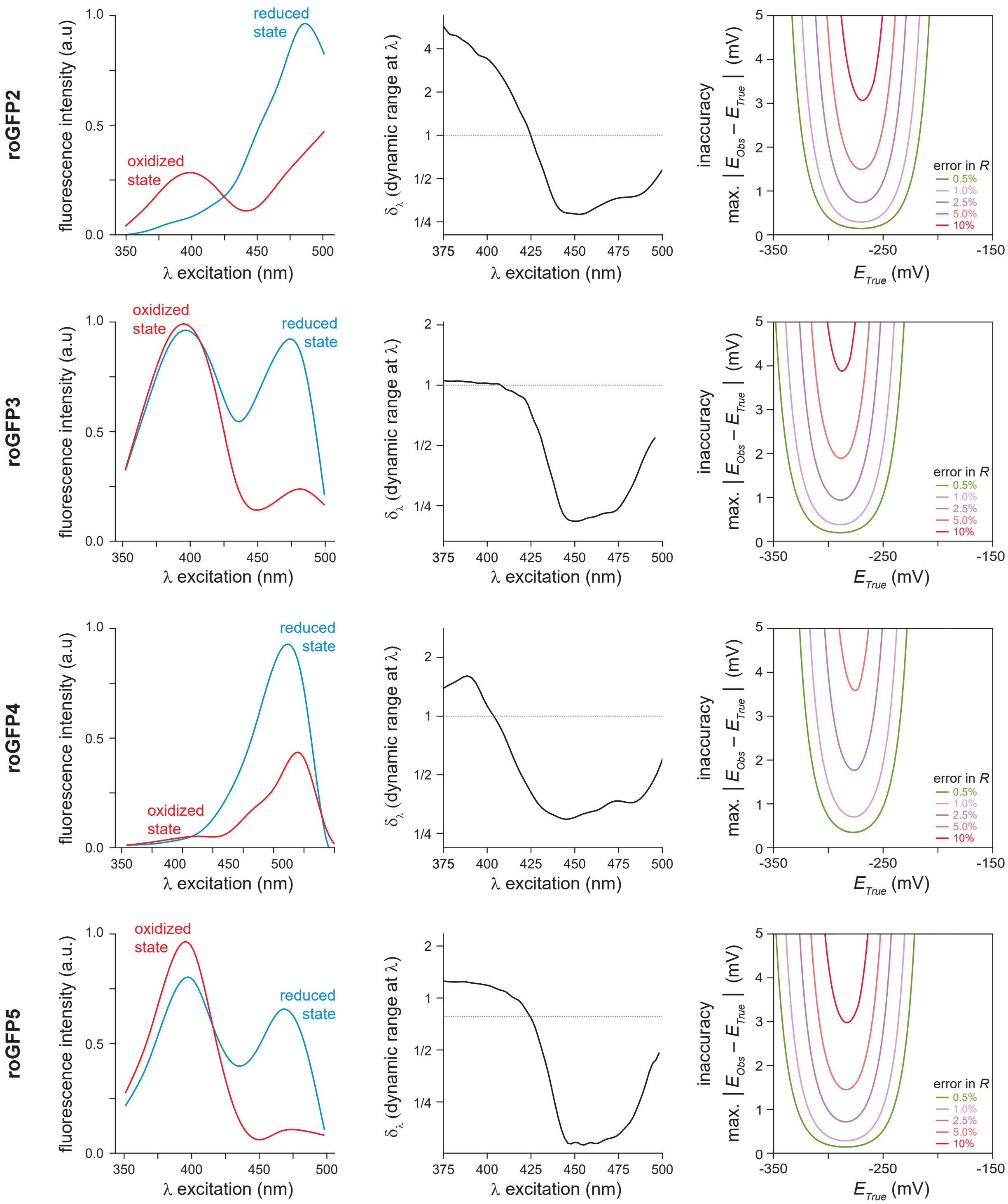

roGFP2
roGFP3
roGFP4
roGFP5
fluorescence intensity (a.u)
fluorescence intensity (a.u.)
λ excitation (nm)
oxidized state
reduced state
δλ (dynamic range at λ)
inaccuracy
max. | EObs − ETrue | (mV)
ETrue (mV)
error in R
0.5%
1.0%
2.5%
5.0%
10%

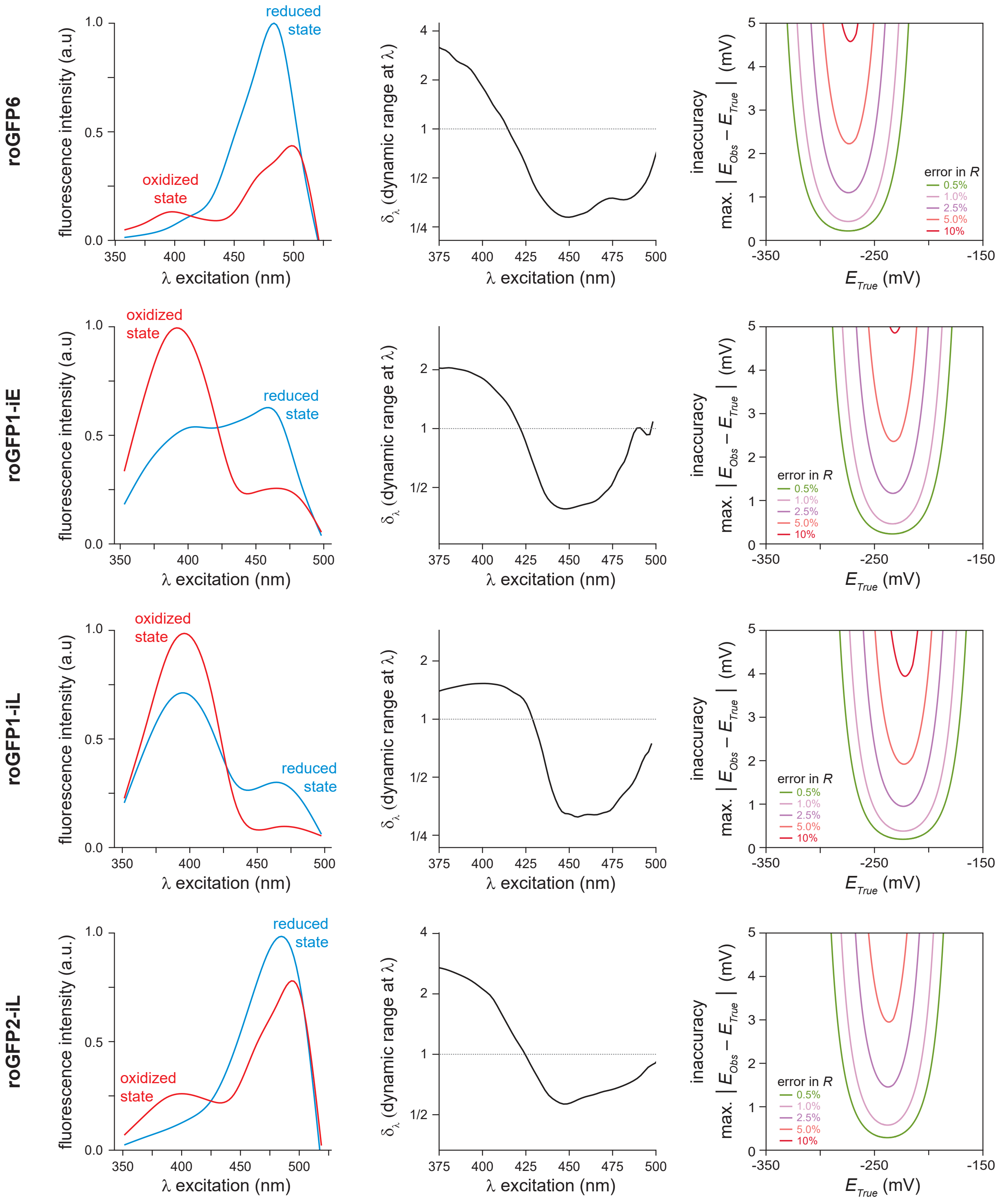
roGFP6
roGFP1-iE
roGFP1-iL
roGFP2-iL
fluorescence intensity (a.u)
fluorescence intensity (a.u.)
λ excitation (nm)
reduced state
oxidized state
δ_λ (dynamic range at λ)
inaccuracy max. | E_Obs − E_True | (mV)
E_True (mV)
error in R
0.5%
1.0%
2.5%
5.0%
10%

Supplementary Note 6

In this note, we compared biochemical and biophysical properties of roGFP biosensors that are important predictors of biosensor E_{GSH} accuracy.

(6.1) Comparing the overall dynamic range and effective midpoint potential of roGFP biosensors

In Supplementary Note 1.3 we derived the function mapping the ratiometric emission (R) of roGFP biosensors to the reduction potential of the glutathione-glutathione disulfide couple E_{GSH}:

$$E_{GSH} = E_{roGFP} = E^{\circ\prime}_{roGFP} - \frac{R_{gas}T}{2F}\ln\left(\delta_{\lambda_2}\frac{R_{ox} - R}{R - R_{red}}\right) \tag{1}$$

In Supplementary Note 4.2 we modeled the sensitivity of E_{GSH} measurement inaccuracy to changes in the values of the parameters in this map that quantify the biochemical and biophysical properties of the biosensor and instrumentation. Changes in two of these parameters shift E_{GSH} by constant amounts: the biosensor's standard midpoint potential $E^{\circ\prime}_{roGFP}$ and the biosensor's dynamic range in the second excitation wavelength δ_{λ_2}. We can aggregate these two effects into a single effective midpoint potential $E^{\circ\prime}_{effective}$:

$$E^{\circ\prime}_{effective} = E^{\circ\prime}_{roGFP} - \frac{R_{gas}T}{2F}\ln\delta_{\lambda_2} \tag{2}$$

$$E_{GSH} = E^{\circ\prime}_{effective} - \frac{R_{gas}T}{2F}\ln\frac{R_{ox} - R}{R - R_{red}} \tag{3}$$

In Supplementary Note 4.2, we also found that the optimal strategy for minimizing E_{GSH} inaccuracy with a given biosensor and error level in R is to select excitation wavelengths that maximize the biosensor's dynamic range DR.

Both $E^{\circ\prime}_{effective}$ and DR depend on the choice of excitation wavelengths $\lambda_1$and λ_2. As a result, the values of these parameters can vary across experimental settings. In Supplementary Note 5.2 we derived the values of DR and δ_{λ_2} for each of the eleven roGFP biosensors with known fluorescence spectra. These values were determined for two illumination settings: one matched the bandwidths of the excitation filters in our microscopy set up and the other matched the

wavelengths of widely used lasers. Using each of those two sets of parameters, we inspected how $E^{\circ\prime}_{effective}$ and DR covary across roGFP biosensors (Figure S6-1). This analysis suggested that no roGFP biosensor had DR values above 5 and $E^{\circ\prime}_{effective}$ values above -275 mV under either imaging modality (Figure S6-1a,b). Most biosensors were predicted to have higher DR values using parameters for 410±15 nm and 470±10 nm illumination (Figure S6-1a) than using parameters for 405 nm and 488 nm illumination (Figure S6-1b).

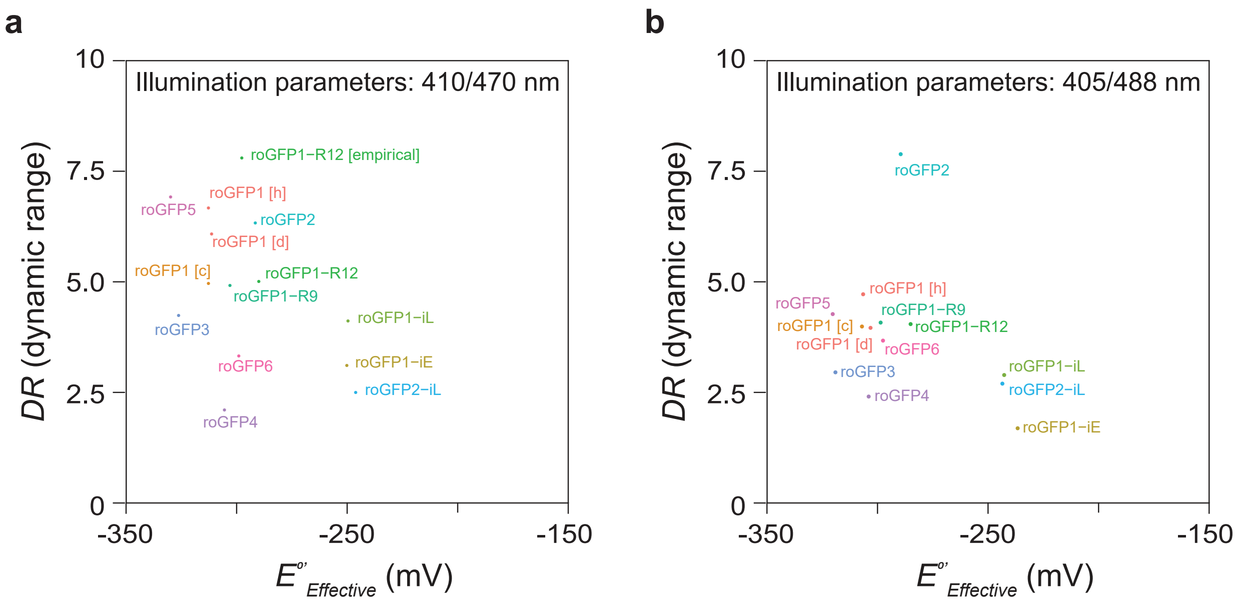

Figure S6-1: $E^{\circ\prime}_{effective}$ and DR covary across roGFP biosensors

The values of $E^{\circ\prime}_{effective}$ and DR depend on the choice of excitation wavelengths $\lambda_1$and λ_2 and therefore can vary across experimental settings. The scatterplots in panels **a** and **b**, show the covariation of $E^{\circ\prime}_{effective}$ and DR of the eleven roGFP biosensors with known fluorescence spectra for two experimental settings. In panel **a**, these values were determined for the illumination setting matching the bandwidths of the excitation filters in our microscopy set up; while, in panel **b**, these values were determined for the illumination setting matching the wavelengths of widely used 405 nm and 488 nm lasers.

Supplementary Note 7

In this note, we build on the framework described in Supplementary Note 1 to derive (7.1) the map from fluorescence-ratio (R) measurements to pH in two-state ratiometric pH biosensors; and (7.2) the more general map from R to ligand concentration in ligand-binding two-state ratiometric biosensors.

(7.1) Mapping *R* to pH in two-state ratiometric pH biosensors

(7.1.1) General chemistry of pH-sensitive two-state ratiometric biosensors

The chromophore of GFP and the chromophores of nearly all fluorescent proteins are formed via a spontaneous series of reactions within an internal X-Tyr-Gly sequence [19, 20]. The phenolic hydroxyl group in the chromophore in each of these proteins, derived from the Tyr side chains, is a weak acid. Protonated and deprotonated chromophore forms exhibit different fluorescence-excitation peaks. For example, in the case of GFP, the protonated chromophore exhibits an excitation peak at 395 nm while the deprotonated form exhibits a peak at 475 nm [2]. The protonation state of the chromophore of many fluorescent proteins, including GFP and mRFP, is relatively insensitive to the pH of the surrounding environment [21]. In contrast, many mutant variants derived from GFP and other fluorescent proteins have chromophores whose protonation state is sensitive to the pH of the surrounding environment and can, therefore, be used as pH biosensors [2].

For many pH biosensors, the protonated chromophore state exhibits very poor fluorescence emission. Therefore, the fluorescence emission of an ensemble of such pH biosensors depends on the number of deprotonated biosensors and the fraction of those biosensors in the deprotonated state. Because both of these quantities are generally unknown *in vivo*, these intensity-based biosensors can be useful reporters of changes in pH but are not useful for absolute pH measurement [20].

Ratiometric pH-sensitive biosensors overcome this limitation, enabling calculation of the fraction of deprotonated biosensors from the ratio of the biosensor's fluorescence excitation and/or emission at two different wavelengths (R), as we describe in Supplementary Note 1.2.

Knowledge of the fraction of deprotonated biosensors is a first step towards measuring pH with pH-sensitive biosensors, which also requires knowledge of the biosensor's tendency to become

protonated and deprotonated. The balance between protonated and deprotonated chromophore forms of a fluorescent biosensor is described by the weak acid equation:

$$[Biosensor^-] + [H^+] \rightleftarrows [BiosensorH] \tag{1}$$

where $BiosensorH$ and $Biosensor^-$ are the biosensors with protonated and deprotonated chromophores, respectively. At equilibrium, the balance among the concentrations of these species is described by the biosensor's chromophore acid dissociation constant K_a:

$$K_a = \frac{[Biosensor^-] \times [H^+]}{[BiosensorH]} \tag{2}$$

$K_a$is determined by the local environment surrounding the chromophore and is therefore a property of each biosensor. Solving for $[H^+]$, we obtain the Henderson-Hasselbach equation:

$$pH = pK_a + log_{10} \frac{[Biosensor^-]}{[BiosensorH]} \tag{3}$$

In the section (7.1.3) we use this equation to calculate pH given knowledge of both the biosensor's $K_a$and the fraction of deprotonated biosensors.

<u>(7.1.2) Deriving the map between a biosensor's fluorescence ratio and the fraction of deprotonated biosensors</u>

The derivation of the map between R and $P(Biosensor^-)$ builds on the framework developed for two-state ratiometric biosensors in Supplementary Note 1.2. For roGFPs, the A and B biosensor states refer to the oxidized and reduced biosensor species. Here, these two states refer to the deprotonated and protonated biosensor species. Starting with equation 12 from Supplementary Note 1.2, and substituting $Biosensor^-$ and $BiosensorH$ for the biosensor's A and B states, we obtain the map between R and $P(Biosensor^-)$:

$$P(Biosensor^-) = \frac{R - R_{BiosensorH}}{R - R_{BiosensorH} + \delta_{\lambda_2}(R_{Biosensor^-} - R)} \tag{4}$$

<u>(7.1.3) Deriving the map between a biosensor's fluorescence ratio and pH</u>

The concentrations of protonated and deprotonated biosensor species are constrained by mass balance:

$$[Biosensor] = [Biosensor^{-}] + [BiosensorH] \tag{5}$$

Substituting the Henderson-Hasselbach equation we obtain:

$$pH = pK_a - log_{10}\frac{1 - P(Biosensor^{-})}{P(Biosensor^{-})} \tag{6}$$

Substituting the map between R and $P(Biosensor^{-})$ we obtain:

$$pH = pK_a - log_{10}(\delta_{\lambda_2}\frac{R_{Biosensor^{-}} - R}{R - R_{BiosensorH}}) \tag{7}$$

This is the map between a biosensor's fluorescence ratio and the pH of the surrounding environment under equilibrium conditions.

We note that this map has the same general form as that from R to E_{GSH} in roGFP biosensors (see Supplementary Note 1.3). In the next section, we extend this framework to ligand-binding biosensors.

(7.2) Mapping *R* to ligand concentration in two-state ratiometric ligand-binding biosensors

7.2.1. General chemistry of ligand-binding biosensors

The protonation-deprotonation reaction of a pH-sensitive biosensor:

$$[Biosensor^{-}] + [H^{+}] \rightleftarrows [BiosensorH] \tag{8}$$

is a special case of the more general binding-unbinding reaction of a single ligand and a single biosensor:

$$[Biosensor] + [Ligand] \rightleftarrows [Biosensor - Ligand] \tag{9}$$

where $[Biosensor]$, $[Ligand]$, and $[Biosensor - Ligand]$ represent the concentrations of unbound free biosensor, unbound free ligand, and biosensor-ligand complex.

At equilibrium, the balance among the concentrations of $Biosensor$, $Ligand$, and $Biosensor - Ligand$ species is described by the dissociation constant K_d:

$$K_d = \frac{[Biosensor] \times [Ligand]}{[Biosensor - Ligand]} \tag{10}$$

In section 7.2.2, we derive the map from a biosensor's fluorescence ratio (*R*) to the concentration of its ligand for two-state ratiometric ligand-binding biosensors.

<u>(7.2.2) Deriving the map between a biosensor's fluorescence ratio and ligand concentration</u>

Specific two-state ratiometric biosensors exist for many ligands, including Histidine, NAD^+, NADH, and NADPH. The derivation of the map between R and $[Ligand]$ mirrors that between R and pH in two-state ratiometric pH biosensors. Starting with equation 12 from Supplementary Note 1.2, and substituting $Biosensor$ and $Biosensor - Ligand$ for the biosensor's A and B states, we obtain the map between R and $P(Biosensor)$:

$$P(Biosensor) = \frac{R - R_{Biosensor-Ligand}}{R - R_{Biosensor-Ligand} + \delta_{\lambda_2}(R_{Biosensor} - R)} \tag{11}$$

Solving for $[Ligand]$ we obtain:

$$[Ligand] = 10^{-pK_d + \, log_{10}\frac{1-P(Biosensor)}{P(Biosensor)}} \tag{12}$$

Substituting the map between R and $P(Biosensor)$ we obtain:

$$[Ligand] = 10^{-pK_d + \, log_{10}(\delta_{\lambda_2}\frac{R_{Biosensor}-R}{R-R_{Biosensor-Ligand}})} \tag{13}$$

This is the map between a biosensor's fluorescence ratio and the free ligand concentration of the surrounding environment under equilibrium conditions.

Supplementary Note 8

In Supplementary Note 7, we derived the functions mapping the ratiometric emission (R) of two-state biosensors to the pH in the environment surrounding those biosensors under equilibrium conditions. Because those maps have the same general form as those from R to E_{GSH} in roGFP biosensors (see Supplementary Note 1.3), they also have a similar dependence on biochemical and biophysical parameters quantifying the properties of the biosensors and the instrumentation used to measure their fluorescence (see Supplementary Note 5.1). Knowledge of those parameters enabled us to model how the precision of R measurements with a biosensor determines the range of E_{GSH} values that is possible to measure with that biosensor at a specific inaccuracy level (see Supplementary Note 5.2 and 5.3). In this note, we build on that scheme to model how the precision of R measurements with a biosensor determines the range of pH values that is possible to measure with that biosensor at a given inaccuracy level. We (8.1) estimate the values of the minimal set of parameters and constraints between parameters needed to predict the influence of relative errors in R on nine pH biosensors with known pK_a and fluorescence spectra; and (8.2) determine the pH inaccuracy we would expect to observe with each of those biosensors if we selected optimal excitation or emission filters for each biosensor and measured R at the same precision as our R measurements in the feeding muscles of live *C. elegans* with roGFP-R12. In Supplementary Note 9 we extend this analysis to two-state ligand-binding ratiometric biosensors.

(8.1) Obtaining the values of the parameters that map R to pH for nine two-state ratiometric pH biosensors

<u>(8.1.1) Overall approach</u>

In Supplementary Note 7.1.3 we derived the function mapping a biosensor's fluorescence ratio (R) and the pH of the environment surrounding the biosensor under equilibrium conditions.

$$pH = pK_a - log_{10}(\delta_{\lambda_2} \frac{R_{Biosensor^-} - R}{R - R_{BiosensorH}}) \quad (1)$$

This map depends on biochemical and biophysical parameters. The biochemical parameter is the biosensor's chromophore acid dissociation constant K_a. This parameter is often known from published reports; however, in many cases the values reported do not represent true K_a values as they are extracted from pH-titrations of the biosensor's fluorescence ratio, which do not necessarily have a linear relationship with the fraction of protonated biosensors (see Supplementary

Note 2). Therefore, we show how these reported effective K_a values can be corrected with knowledge of some of the biosensor's biophysical parameters (section 8.1.2).

The biophysical parameters required to map R to pH include the biosensor's dynamic range in the second wavelength δ_{λ_2}, the ratiometric emission of an ensemble of protonated biosensors $R_{BiosensorH}$, and the ratiometric emission of an ensemble of deprotonated biosensors $R_{Biosensor^-}$. These three parameters can be determined empirically, but in most cases they are not known. The value of δ_{λ_2} and the biosensor's overall dynamic range DR (equal to the $R_{Biosensor^-}/R_{BiosensorH}$ ratio) can be determined from the fluorescence spectra of protonated and deprotonated biosensors, similar to the case of reduced and oxidized biosensor species (see Supplementary Note 5.1.2). In the case of pH biosensors, however, published reports often underestimate the biosensor's dynamic range because these reports only include the spectra of biosensor species that are not fully protonated or fully deprotonated, often because the biosensors begin to unfold at pH > 10 [3]. We show how these published spectra and knowledge of the biosensor's K_a value can be used to estimate the spectra of protonated and deprotonated biosensor species, leading to more accurate estimates of the biosensor's dynamic range at each wavelength (section 8.1.3).

We then estimate the values of the minimal set of parameters and constraints between parameters needed to predict the influence of relative errors in R on nine pH biosensors with known pK_a and fluorescence spectra (section 8.1.4). As discussed in Supplementary Note 5.1.3 for the map from R to E_{GSH}, mapping the R values obtained with a given instrument to pH requires knowledge of the values of both $R_{BiosensorH}$ and $R_{Biosensor^-}$ in that instrument; however, when all other parameters are known, knowledge of the $R_{Biosensor^-}/R_{BiosensorH}$ ratio is sufficient for modeling how relative errors in R would influence the expected pH inaccuracy of different biosensors under otherwise identical microscopy conditions.

<u>(8.1.2) Obtaining the pKa of a biosensor from a pH-titration of its fluorescence ratio</u>

The balance among the concentrations of protonated and deprotonated species of a pH biosensor is quantified by the biosensor's chromophore acid dissociation constant K_a. Empirically, K_a is often determined as the point in a pH titration where deprotonated and protonated biosensor species have equal concentrations. However, studies often report the pH value where the biosensor's fluorescence ratio is half-way between the values corresponding to protonated and deprotonated

biosensors. These "effective pK_a" ($pK_{effective}$) values do not represent true pK_a values as they are extracted from pH-titrations of the biosensor's fluorescence ratio, and not of the concentration of specific biosensor species. To convert into pK_a values the reported $pK_{effective}$ values, we substitute R = $(R_{Biosensor^-} + R_{BiosensorH})/2$ in equation 1:

$$pH = pK_{effective} = pK_a - log_{10}\delta_{\lambda_2} \quad (2)$$

where δ_{λ_2} is the biosensor's dynamic range in the second wavelength used in the titration. Therefore:

$$pK_a = pK_{effective} + log_{10}\delta_{\lambda_2} \quad (3)$$

(8.1.3) Obtaining δ_{λ_2} and DR from the excitation spectra of pH biosensor species that are not fully protonated or deprotonated

Published reports often only include the spectra of biosensor species that are not fully protonated or fully deprotonated and therefore underestimate the biosensor's dynamic range. Here, we show how to estimate the biosensor's dynamic range using published spectra obtained at two pH values and knowledge of the biosensor's pK_a value.

In Supplementary Note 1.2.1, we showed that in a mixed population of biosensors the total observed fluorescence intensity will be the weighted average of the intensities of populations of biosensors in each state:

$$I_\lambda = P(A)\, I_{\lambda,A} + [1 - P(A)]\, I_{\lambda,B} \quad (4)$$

Where λ represents an excitation- and emission-wavelength pair, A and B are the two biosensor states, $P(A)$ is the fraction of biosensors in state A, and $I_{\lambda,A}$ and $I_{\lambda,B}$ are, respectively, the total observed intensities of ensembles of biosensors in states A and B. For pH biosensors, the A state corresponds to the deprotonated state and the B state corresponds to the protonated state.

To obtain $I_{\lambda,Biosensor^-}$ and $I_{\lambda,BiosensorH}$ from I_λ measurements at two different pH values, x and y, we first calculate $P(Biosensor^-)$ at those pH values:

$$a = P(Biosensor^{-}, pH = x) = \frac{1}{1 + 10^{(pK_a - x)}} \tag{5}$$

$$b = P(Biosensor^{-}, pH = y) = \frac{1}{1 + 10^{(pK_a - y)}} \tag{6}$$

Substituting equations 5 and 6 in equation 4, we obtain:

$$I_{\lambda,x} = \ a\, I_{\lambda,Biosensor^{-}} + (1 - a)\, I_{\lambda,BiosensorH} \tag{7}$$

$$I_{\lambda,y} = \ b\, I_{\lambda,Biosensor^{-}} + (1 - b)\, I_{\lambda,BiosensorH} \tag{8}$$

Therefore:

$$\frac{I_{\lambda,x} - a\, I_{\lambda,Biosensor^{-}}}{1 - a} = \frac{I_{\lambda,y} - b\, I_{\lambda,Biosensor^{-}}}{1 - b} \tag{9}$$

$$\frac{I_{\lambda,x} - (1 - a)\, I_{\lambda,BiosensorH}}{a} = \frac{I_{\lambda,y} - (1 - b)\, I_{\lambda,BiosensorH}}{b} \tag{10}$$

Solving for $I_{\lambda,Biosensor^{-}}$ and $I_{\lambda,BiosensorH}$:

$$I_{\lambda,Biosensor^{-}} = \frac{(1 - b)\, I_{\lambda,x} - (1 - a)\, I_{\lambda,y}}{a - b} \tag{11}$$

$$I_{\lambda,BiosensorH} = \frac{b\, I_{\lambda,x} - a\, I_{\lambda,y}}{b - a} \tag{12}$$

(8.1.4) pK_a, δ_{λ_1}, δ_{λ_2}, $R_{BiosensorH}$ and $R_{Biosensor^-}$, and DR parameter values for nine two-state ratiometric pH biosensors

For fifteen ratiometric two-state pH biosensors, we attempted to obtain the biosensor's pK_a value and the fluorescence excitation spectra of protonated and deprotonated biosensor species from published data (Table S8-1). Of the five biosensors where the pK_a was unknown, the $pK_{effective}$ was known for two, Sypher and Sypher3s. For these biosensors, we obtained an estimate of their pK_a from pH-titrations of their fluorescence ratios, following the scheme described in section 8.1.2 (Table S8-2). The fluorescence excitation spectra of protonated and deprotonated biosensor species have not been reported for four of the fifteen pH biosensors (Table S8-1).

For the nine biosensors with available spectra and known or estimated pK_a, spectra collected at different pH values were digitized with the WebPlotDigitizer software [10], and the spectra of fully protonated and fully deprotonated biosensor states were estimated following the scheme described in section 8.1.3. In nine cases these spectra differed only slightly from the empirical measurements (Figures S8-1,2,3), because these measurements had been obtained at pH values at least one pH unit above and one pH unit below the pK_a of the biosensor. The exception was SypHer, a biosensor where all spectra were collected at pH values below the pK_a. The estimate of this biosensor's $\delta_{\lambda_2}$and DR were therefore considered of insufficient quality and the biosensor was not studied further.

For each of the eight remaining pH biosensors, δ_{λ_1}, δ_{λ_2}, $R_{BiosensorH}$, $R_{Biosensor^-}$, and DR parameter values were collected from the estimated fluorescence intensity spectra of fully protonated and fully deprotonated biosensor states (Table S8-2). For each biosensor, we chose excitation-emission-wavelength pairs, λ_1 and λ_2, seeking to maximize DR and to match the wavelengths at which the biosensor's fluorescence intensity peaked. We used fluorescence intensity signal averaged over 20 nm intervals centered around λ_1 and λ_2 to calculate δ_{λ_1}, δ_{λ_2}, $R_{BiosensorH}$, $R_{Biosensor^-}$, and DR. The values of these parameters for each biosensor were determined as described in (8.1). These parameters varied significantly between biosensors (Table S8-2 and Figures S8-1,2,3 second column).

(8.2) Predicted pH inaccuracy of eight ratiometric two-state pH biosensors

Using the pK_a, δ_{λ_2}, $R_{BiosensorH}$, and $R_{Biosensor^-}$ parameters obtained in (8.1), we mapped R to pH for eight ratiometric two-state pH biosensors. We applied the SensorOverlord framework to

determine the pH inaccuracy that we would expect to observe at each pH value with each biosensor, given our empirical distribution of relative errors in R when measuring E_{GSH} in the feeding muscles of live *C. elegans* expressing roGFP-R12. This analysis was applied to dual-excitation red-fluorescent pH biosensors (Figure S8-1, third column), dual-excitation green-fluorescent pH biosensors (Figure S8-2, third column), and single-excitation dual-emission biosensors (Figure S8-3, third column). The E^2GFP biosensor can be used in two different modalities, dual-excitation green-fluorescence and single-excitation dual-emission. The differences in the biosensor's δ_{λ_2} and DR parameter values in each imaging modality lead to differences in the predicted pH inaccuracy of this biosensor under each imaging modality.

Table S8-1: two-state ratiometric pH biosensors

Biosensor		pK_a	$pK_{effective}$		Spectra?	
deGFP1	[3]	8.02		[3]	Yes	[3, 13]
deGFP2	[3]	7.25		[3]	No	
deGFP3	[3]	6.86		[3]	No	
deGFP4	[3]	7.37		[3]	Yes	[3, 13]
ClopHensor	[22]	6.78		[22]	Yes	[22]
E^1GFP	[23]	No			No	
E^2GFP	[24]	7.01<br>6.78		[23, 24]	Yes	[24]
ratiometric-pHluorin	[25]	No			Yes	[25]
pHluorin2	[26]	No			Yes	[26]
mCherryEA	[27]	7.8		[27]	Yes	[27]
pHRed	[28]	7.8		[28]	Yes	[28]
mKeima	[28]	7.8		[28]	Yes	[28]
SypHer3s	[29]	No	7.8	[29]	Yes	[29]
SypHer-2	[30]	8.1		[30]	No	
SypHer	[31]	No	8.71	[31]	Yes	[31]

Table S8-2: Biosensor parameter values used for mapping R to pH

Biosensor	Imaging modality	$\lambda_1 \pm 10\ nm$	$\lambda_2 \pm 10\ nm$	pK_a	DR	δ_{λ_1}	δ_{λ_2}	$R_{BiosensorH}$	$R_{Biosensor^-}$
mCherryEA	dual excitation	455	585	7.8	9.87	3.16	0.32	0.30	2.96
pHRed	dual excitation	440	585	7.8	62.6	6.26	0.10	0.15	9.39
mKeima	dual excitation	440	560	7.8	21.3	2.13	0.10	0.95	20.2
E^2GFP	dual excitation	480	440	6.78	15.7	1.26	0.08	0.32	5.02
ClopHensor	dual excitation	488	430	6.78	8.64	2.85	0.33	1.16	10.0
SypHer3s	dual excitation	495	410	8.5 *	414	53.9	0.13	0.19	78.7
E^2GFP	dual emission	525	505	6.78	5.02	4.17	0.83	0.82	4.12
deGFP4	dual emission	515	460	7.37	62.5	10.0	0.16	0.47	29.4
deGFP1	dual emission	515	460	8.02	47.3	10.9	0.23	0.58	27.4

* pK_a derived from $pK_{effective}$ as described in section 8.1.4.

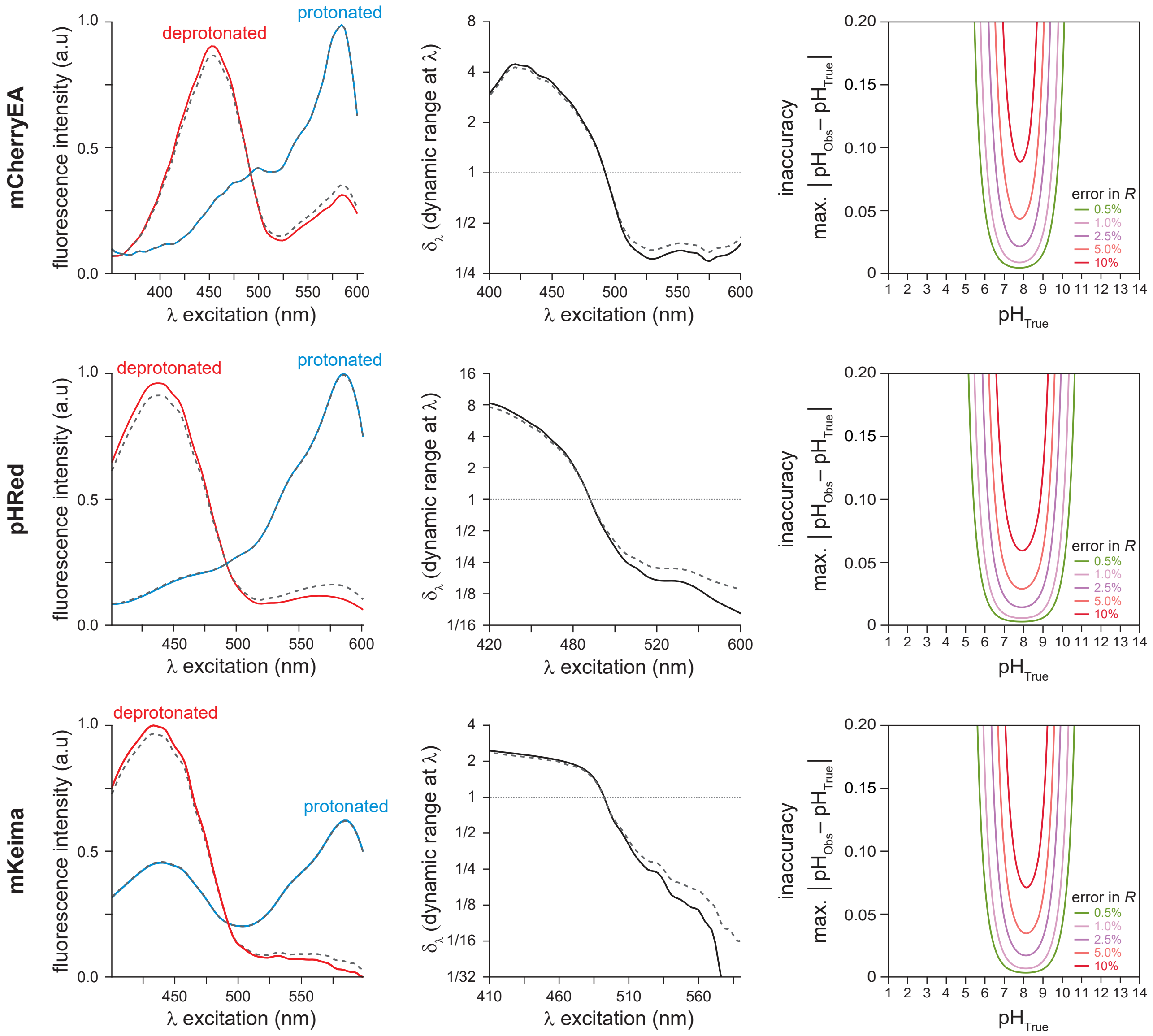

Figure S8-1: Predicted pH inaccuracy of dual-excitation red-fluorescent pH biosensors

In this analysis of three dual-excitation red-fluorescent pH biosensors, we show in each row a plot of the biosensor's predicted emission when excited at a wavelength λ in the fully deprotonated and protonated states (solid lines, column 1) and the observed emissions of mostly deprotonated and mostly protonated biosensor mixtures (dotted lines, column 1); a plot of the biosensor's dynamic range δ_λ, the predicted ratio of the emissions of the fully deprotonated and protonated biosensor states (solid line, column 2) and the apparent δ_λ derived from the ratio of the observed emissions of mostly deprotonated and mostly protonated biosensor mixtures (dotted line, column 2); and a plot of the predicted pH measurement inaccuracy with that biosensor at each wavelength λ for different relative errors in R values (column 3).

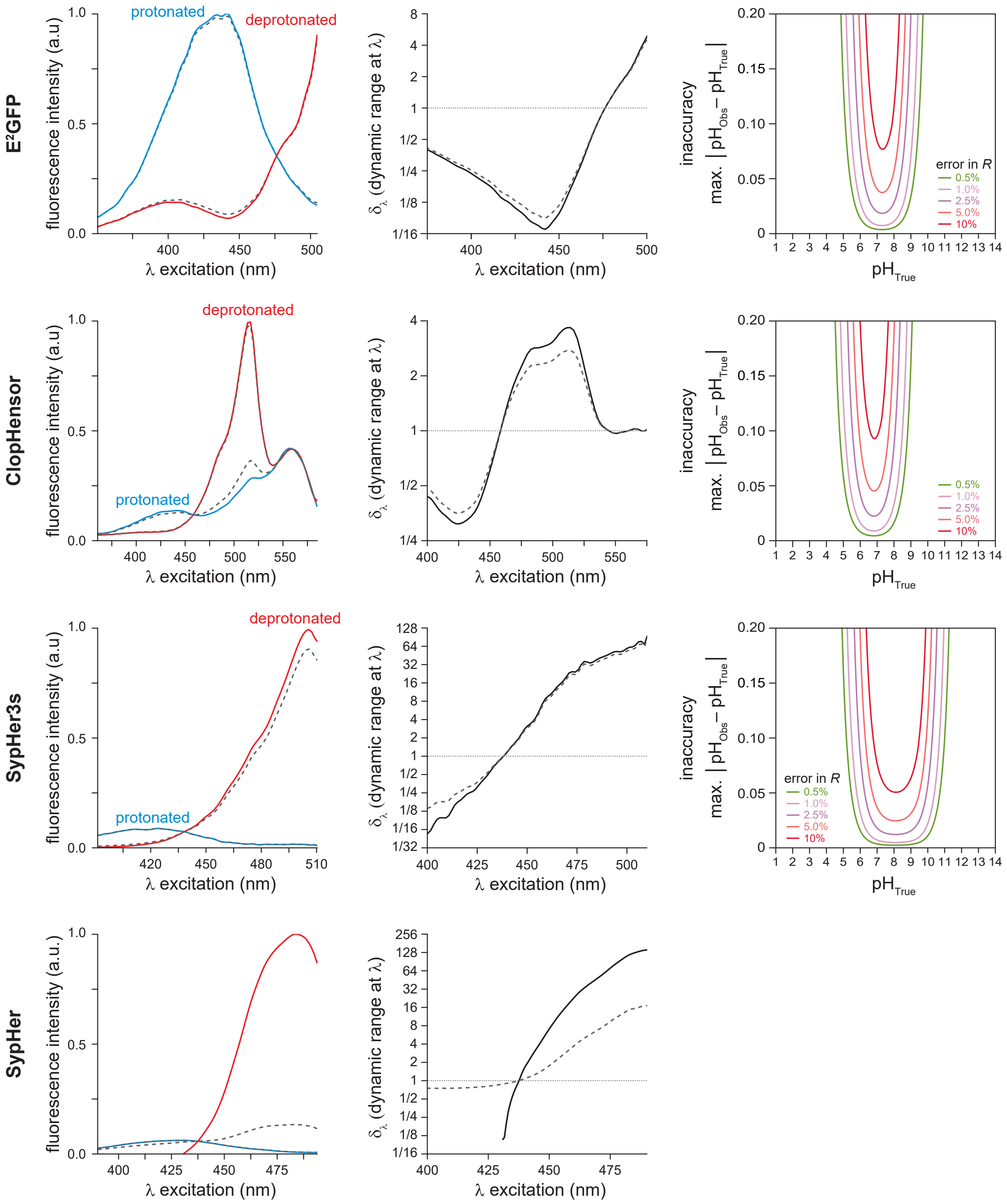

Figure S8-2: Predicted pH inaccuracy of dual-excitation green-fluorescent pH biosensors

In this analysis of four dual-excitation green-fluorescent pH biosensors, we show in each row a plot of the biosensor's predicted emission when excited at a wavelength λ in fully deprotonated

and protonated states (solid lines, column 1) and the observed emissions mostly deprotonated and mostly protonated biosensor mixtures (dotted lines, column 1); a plot of the biosensor's dynamic range δ_λ, the predicted ratio of the emissions of fully deprotonated and protonated biosensor states (solid line, column 2) and the apparent δ_λ derived from the ratio of the observed emissions of mostly deprotonated and mostly protonated biosensor mixtures (dotted line, column 2); and a plot of the predicted pH measurement inaccuracy with that biosensor at each wavelength λ for different relative errors in R values (column 3).

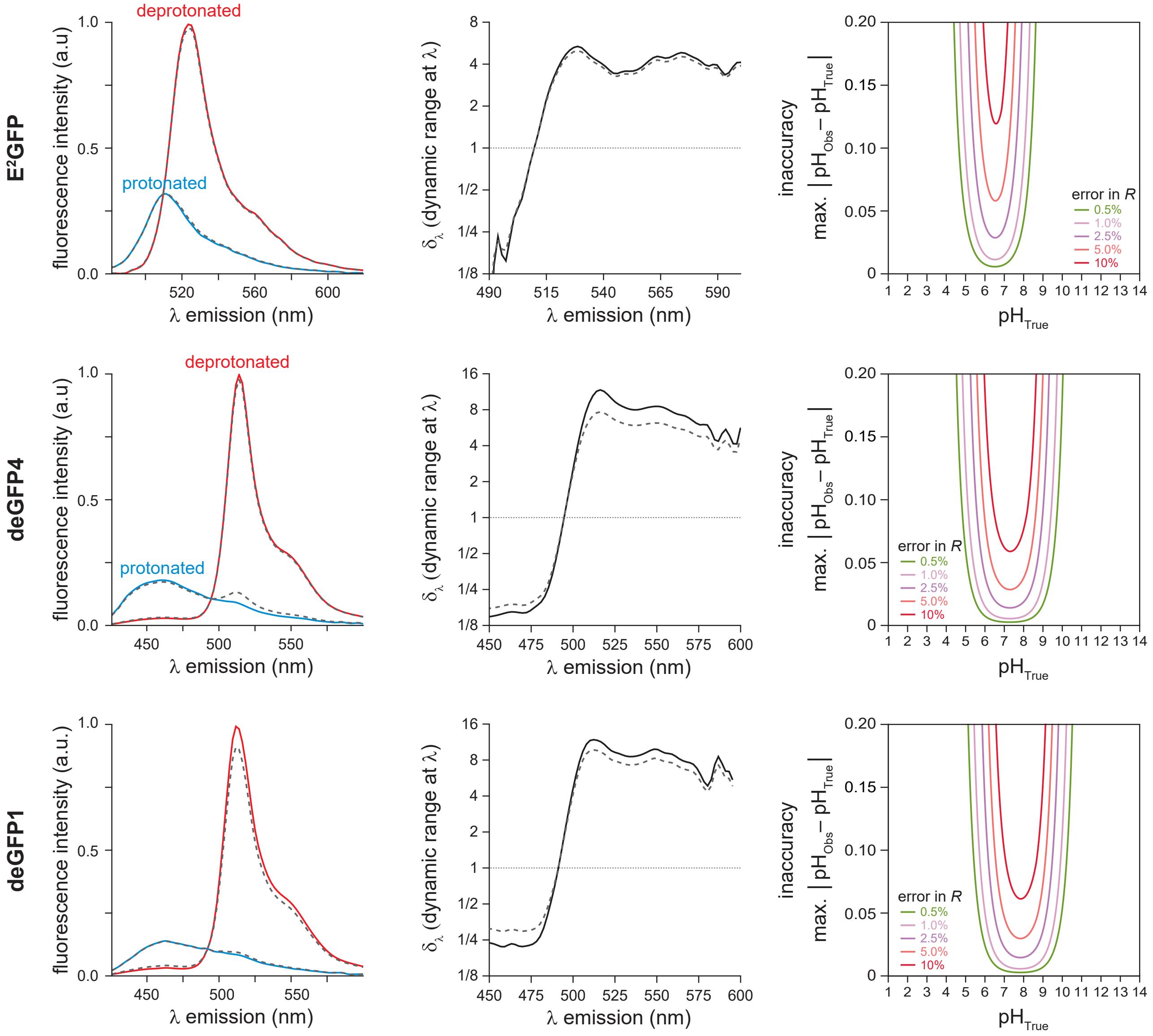

Figure S8-3: Predicted pH inaccuracy of single-excitation dual-emission pH biosensors

In this analysis of three single-excitation dual-emission pH biosensors, we show in each row a plot of the biosensor's predicted emission when excited at a wavelength λ in fully deprotonated and protonated states (solid lines, column 1) and the observed emissions mostly deprotonated and mostly protonated biosensor mixtures (dotted lines, column 1); a plot of the biosensor's dynamic range δ_λ, the predicted ratio of the emissions of fully deprotonated and protonated biosensor states (solid line, column 2) and the apparent δ_λ derived from the ratio of the observed emissions of mostly deprotonated and mostly protonated biosensor mixtures (dotted line, column 2); and a plot of the predicted pH measurement inaccuracy with that biosensor at each wavelength λ for different relative errors in R values (column 3).

Supplementary Note 9

In Supplementary Note 7, we derived the functions mapping the ratiometric emission (R) of ligand-binding two-state biosensors to their ligand concentration in their surrounding environment under equilibrium conditions. These maps follow the same form as those mapping R to pH in two-state ratiometric pH biosensors. This enables us, in this note, to follow the scheme used in Supplementary Note 8, to model how the precision of R measurements with a ligand-binding biosensor determines the range of ligand concentration values that is possible to measure with that biosensor at a specific inaccuracy level. We (9.1) estimate the values of the minimal set of parameters and constraints between parameters needed to predict the influence of relative errors in R on ligand-binding biosensors with known K_d and fluorescence spectra; and (9.2) determine the ligand-concentration inaccuracy we would expect to observe with each of those biosensors if we selected optimal excitation or emission filters for each biosensor and measured R at the same precision as our R measurements in the feeding muscles of live *C. elegans* with roGFP-R12.

(9.1) Obtaining the values of the parameters that map R to ligand concentration for ligand-binding biosensors

(9.1.1) Overall approach

In Supplementary Note 7.2.2 we derived the function mapping a biosensor's fluorescence ratio (R) and the free ligand concentration in the environment surrounding the biosensor under equilibrium conditions.

$$[Ligand] = 10^{-pK_d + log_{10}(\delta_{\lambda_2}\frac{R_{Biosensor}-R}{R-R_{Biosensor-Ligand}})} \quad (1)$$

which can be rewritten as:

$$p[Ligand] = pK_d - log_{10}(\delta_{\lambda_2}\frac{R_{Biosensor}-R}{R-R_{Biosensor-Ligand}}) \quad (2)$$

This function has the same form as the function mapping R and pH (see Supplementary Note 7.1.3) and therefore depends on equivalent biochemical and biophysical parameters. The biochemical parameter is the biosensor's dissociation constant K_d. This parameter is often known from published reports; however, in many cases the values reported do not represent true K_d values as they are extracted from ligand-titrations of the biosensor's fluorescence ratio, which do

not necessarily have a linear relationship with the fraction of ligand-bound biosensors (see Supplementary Note 2). Therefore, we corrected those effective K_d values with knowledge of some of the biosensor's biophysical parameters, following the scheme described for pH biosensors in Supplementary Note 8.1.2.

Published reports often include the spectra only of biosensor species that are not fully ligand-bound and therefore underestimate the biosensor's dynamic range. We estimated biosensor dynamic range using published spectra obtained at two ligand concentrations and knowledge of the biosensor's pK_d value, following the scheme described for pH biosensors in Supplementary Note 8.1.3.

We estimated the values of the minimal set of parameters and constraints between parameters needed to predict the influence of relative errors in R on six ligand-binding biosensors with known pK_d and fluorescence spectra (section 9.1.2). The value of δ_{λ_2} and the biosensor's overall dynamic range DR (equal to the $R_{Biosensor}/R_{Biosensor-Ligand}$ ratio) was determined from the fluorescence spectra of ligand-bound and unbound biosensors. As discussed in Supplementary Note 5.1.3 for the map from R to E_{GSH}, mapping the R values obtained with a given instrument to $p[Ligand]$ requires knowledge of the values of both $R_{Biosensor-Ligand}$ and $R_{Biosensor}$ in that instrument; however, when all other parameters are known, knowledge of the $R_{Biosensor}/R_{Biosensor-Ligand}$ ratio is sufficient for modeling how relative errors in R would influence the expected $p[Ligand]$ inaccuracy of different biosensors, under otherwise identical microscopy conditions.

<u>(9.1.2) pK_d, δ_{λ_1}, δ_{λ_2}, $R_{Biosensor-Ligand}$ and $R_{Biosensor}$, and DR parameter values for six two-state ratiometric ligand-binding biosensors</u>

We attempted to obtain the biosensor's pK_d value and the fluorescence excitation spectra of ligand bound and unbound biosensor species from published data for two-state ratiometric ligand-binding biosensors specific for histidine, NAD^+, NADH, and NADPH (Table S9-1). Of the nine ratiometric two-state ligand-binding biosensors, four had known pK_d values (Table S9-1) and one had a known $pK_{effective}$ from which we estimated pK_d, following the scheme described in section 8.1.2 (Table S9-2). The fluorescence spectra of ligand-bound and unbound biosensor species have not been reported for four of the five iNAP biosensors (Table S9-1), including the iNAP1-

mCherry biosensor. For that biosensor, we assumed iNAP1 NADPH binding affinity and fluorescence emission at 535 nm was unaffected by fusion to mCherry.

For the five biosensors with available spectra and known or estimated pK_d (FHisJ, NAD^+ Biosensor, Frex, FrexH, iNAP1), spectra collected at different ligand concentrations were digitized with WebPlotDigitizer software [10], and the spectra of fully ligand-bound and fully unbound biosensor states were estimated following the scheme described in section 8.1.3. In all cases these spectra differed only slightly from the empirical measurements (Figure S9-1, first column).

We note that because these five biosensors consist of fusions of ligand-binding domains to circularly permuted fluorescent proteins (cpFPs), their spectral properties inherit the pH-dependence of their cpFPs domains. Typically, fluorescence in the first excitation band is pH-resistant, while fluorescence in the second excitation band is pH-sensitive (but with pK_a insensitive to ligand binding). Parameter values were calculated from spectra acquired at a pH of 7.4 (Table S9-1). The iNAP1-mCherry biosensor allows ratiometric and pH-resistant measurement when using the pH-resistant 420 nm excitation band of iNAP1 [32], since mCherry red fluorescence is pH-resistant over a broad range of pH values because it has a pK_a of less than 4.5 [33].

Parameter values for δ_{λ_1}, δ_{λ_2}, $R_{Biosensor-Ligand}$, $R_{Biosensor}$, and DR were collected from the estimated fluorescence intensity spectra of fully ligand-bound and unbound biosensor states (Table S9-2). For each biosensor, we chose excitation- emission-wavelength pairs, λ_1 and λ_2, seeking to maximize DR and to match the wavelengths at which biosensor fluorescence intensity peaked. We used biosensor fluorescence signals averaged over 20 nm intervals centered around λ_1 and λ_2 to calculate δ_{λ_1}, δ_{λ_2}, $R_{Biosensor-Ligand}$, $R_{Biosensor}$, and DR values. The values of these parameters of each biosensor were determined as described in (9.1.2). These parameters varied significantly between biosensors (Table S9-2 and Figures S9-1 second column).

(9.2) Predicted $p[Ligand]$ inaccuracy of six ratiometric two-state ligand-binding biosensors

Using the pK_d, δ_{λ_2}, $R_{Biosensor-Ligand}$, and $R_{Biosensor}$ parameters obtained in (9.1.2), we mapped R to $p[Ligand]$ for six ratiometric two-state ligand-binding biosensors. We applied the SensorOverlord framework to determine the $p[Ligand]$ inaccuracy that we would expect to observe at each $p[Ligand]$ value with each of those biosensors, given our empirical distribution of relative

errors in R when measuring E_{GSH} in the feeding muscles of live *C. elegans* expressing roGFP-R12 (Figure S9-1, third column).

Table S9-1: Two-state ratiometric ligand-binding biosensors

Ligand	Biosensor		pK_d	$pK_{effective}$		Spectra?	
Histidine	FHisJ	[34]	4.66		[34]	Yes	[34]
NAD^+	NAD^+ Biosensor	[35]	4.19		[35]	Yes	[35]
NADH	Frex	[36]		5.43	[36]	Yes	[36]
NADH	FrexH	[36]	7.40		[36]	Yes	[36]
NADPH	iNAP1	[32]	5.70		[32]	Yes	[32]
NADPH	iNAP2	[32]	5.22		[32]	No	
NADPH	iNAP3	[32]	4.60		[32]	No	
NADPH	iNAP4	[32]	3.92		[32]	No	
NADPH	iNAP1-mCherry	[32]	5.70 *		[32]	Inferred †	[32]

* We assumed iNAP1 NADPH binding affinity was unaffected by fusion to mCherry.

† We assumed iNAP1 flurescence emission at 535 nm was unaffected by fusion to mCherry.

Table S9-2: Biosensor parameter values used for mapping R to ligand concentration

Biosensor	Imaging modality	$\lambda_1 \pm 10\ nm$	$\lambda_2 \pm 10\ nm$	pK_d	DR	δ_{λ_1}	δ_{λ_2}	$R_{Bios-Ligand}$	$R_{Biosensor}$
FHisJ	dual excitation	415	485	4.66	6.05	2.06	0.34	0.32	1.93
NAD^+ Bio-sensor	dual excitation	500	425	4.19	1.84	1.86	1.01	2.04	3.76
Frex	dual excitation	410	500	5.45	4.84	0.93	0.19	0.15	0.73
FrexH	dual excitation	500	410	7.40	1.65	2.00	1.22	0.92	1.51
iNAP1	dual excitation	500	420	5.70	7.45	2.13	0.29	0.26	1.93
iNAP1-mCherry	dual excitation / dual emission	560 / 630	420 / 535	5.70 *	3.5 †	1 †	0.29 †	1 †	0.29 †

* We assumed iNAP1 NADPH binding affinity was unaffected by fusion to mCherry.

† We assumed iNAP1 flurescence emission at 535 nm was unaffected by fusion to mCherry.

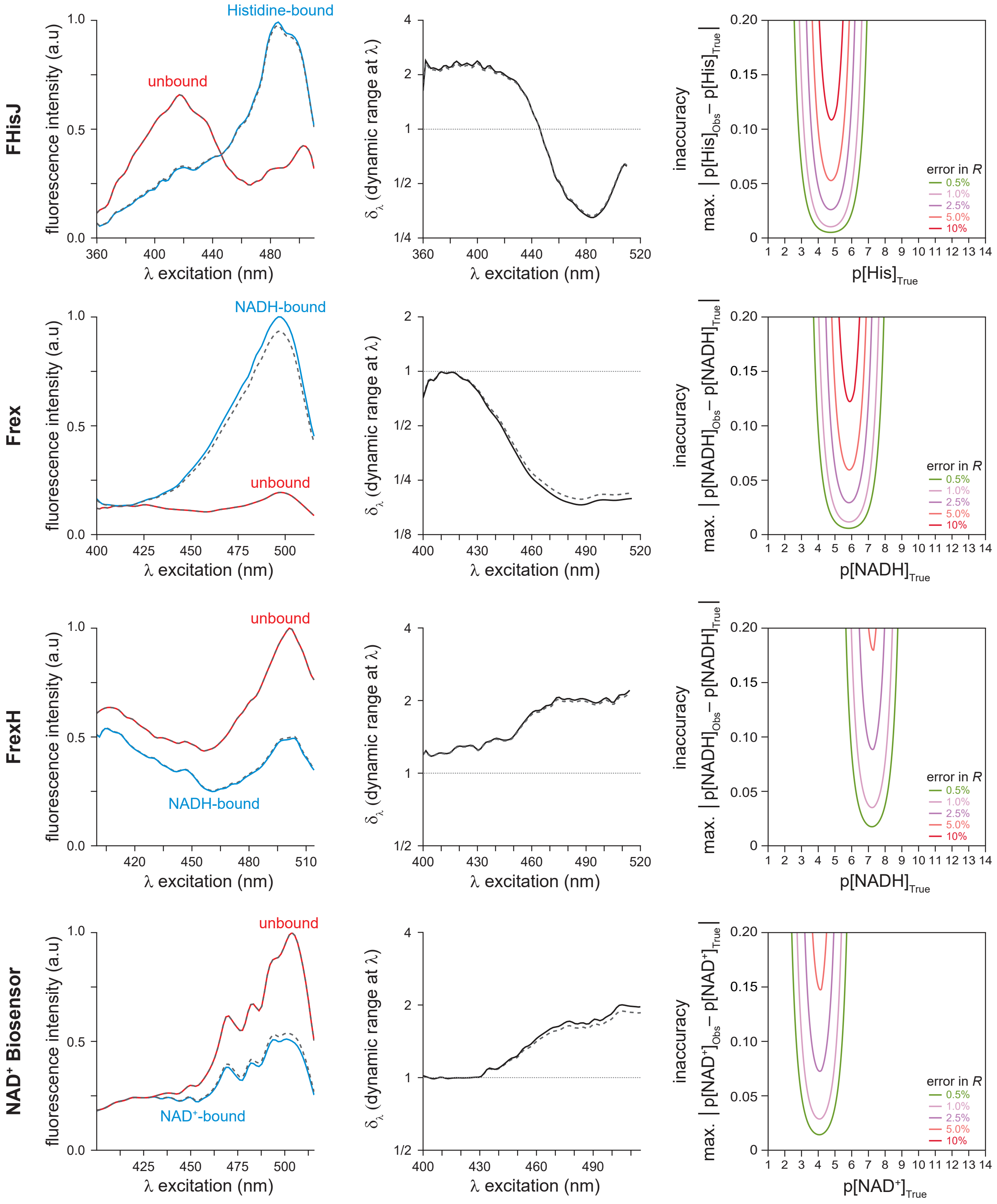

Figure S9-1: Predicted $p[Ligand]$ inaccuracy of ratiometric two-state ligand-binding biosensors

In this analysis of five dual-excitation ligand-binding biosensors, and one dual-excitation dual-emission ligand-binding biosensor, we show in each row a plot of the biosensor's predicted emis-

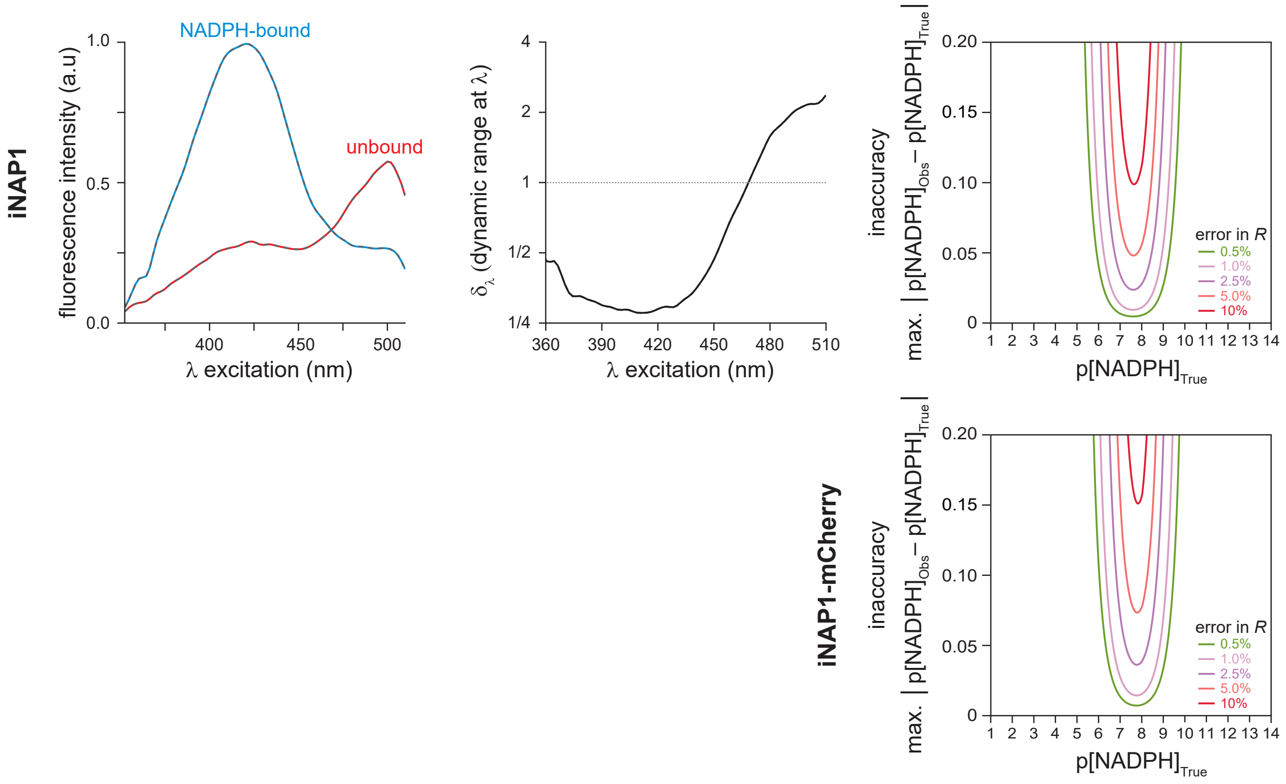

sion when excited at a wavelength λ in the fully ligand-bound and unbound biosensor states (solid lines, column 1) and the observed emissions of mostly ligand-bound and mostly unbound biosensor mixtures (dotted lines, column 1); a plot of the biosensor's dynamic range δ_λ, the predicted ratio of the emissions of the fully ligand-bound and unbound biosensor states (solid line, column 2) and the apparent δ_λ derived from the ratio of the observed emissions of mostly ligand-bound and mostly unbound biosensor mixtures (dotted line, column 2); and a plot of the predicted $p[Ligand]$ measurement inaccuracy with that biosensor at each wavelength λ for different relative errors in R values (column 3).

Supplementary Note 10

In this note we provide a brief user guide to the SensorOverlord R package and web application. We (10.1) provide instructions for installing the R package, (10.2) explain how to obtain each of the parameters required to run the analysis, (10.3) give an example of a use-case for running the SensorOverlord model via the R package, and (10.4) give an example of the use-case for running the SensorOverlord model via the web application. Much of the information provided in this Supplementary Note is also available as vignettes within the SensorOverlord documentation, located at http://apfeldlab.github.io/SensorOverlord/articles.

(10.1) Installing the SensorOverlord R package

We have built an R package, named `sensorOverlord`, that is available on GitHub.

You can install `sensorOverlord` in R via the `devtools` package. To install `devtools`:

```
install.packages("devtools")
```

Then, to install the most recent version of `sensorOverlord` from GitHub:

```
devtools::install_github("apfeldlab/sensorOverlord")
```

To confirm the version of your installation:

```
packageVersion("sensorOverlord")
#> [1] '0.2.0'
```

(10.2) Obtaining requisite parameters

The SensorOverlord framework requires biochemical and biophysical parameters of the ratiometric sensor for which it predicts accuracy. It also requires an empirically-determined error in *R*. In this section, we give an overview of the different methods by which you can obtain these requisite parameters.

<u>(10.2.1) Biochemical parameters</u>

To use the SensorOverlord framework, you need to have access to a midpoint potential for your sensor. For redox biosensors, the midpoint potential is $E^{0'}$. For pH biosensors, the pK_a is used instead of a midpoint potential. For ligand-binding biosensors, the pK_d is used instead of a

midpoint potential. The value of this parameter is almost always reported in the paper in which the sensor of interest was first published.

Note that, for ligand-binding sensors, papers often report K_d. $pKd = -log_{10}(K_d)$ so, to convert K_d to pK_d, use the `log10` function in R:

```
kD <- 5*10e-6
pKd <- -log10(kD)
```

<u>(10.2.2) Biophysical parameters</u>

The SensorOverlord framework requires the following biophysical parameters:

(i) R_A: The ratio measurement of the sensor in State A. We also assume that State A is the state in which you have the lowest ratio measurement, so this is sometimes also called R_{min}.

(ii) R_B: The ratio measurement of the sensor in State B. Sometimes also called R_{max}.

(iii) $\delta_{\lambda 2}$: The dynamic range in the second wavelength. This can be derived from the ratio of spectra of the sensor in each state.

You can obtain these parameters in one of three ways:

(i) Upload a digitized spectrum. `sensorOverlord` can programmatically obtain these input values from a digitized spectrum.

(ii) Use a provided spectrum. We have created a MongoDB database containing the spectra of many published redox, pH, and ligand-binding sensors.

(iii) Provide the parameters directly. These parameters can be measured empirically, as described previously [6].

In the following sections, we elaborate on each of these three options.

<u>(10.2.2.1) Option 1: upload a digitized spectrum</u>

If you have obtained excitation-emission spectrum data for each of your sensor’s two states, you can upload those into R.

For example, you can provide a .csv file with three columns:

- lambda: The wavelength of the excitation or emission.
- values_A: The intensity value of the sensor's output in state A.
- values_B: The intensity value of the sensor's output in state B.

Oftentimes with digitized spectra, the λ values recorded in states A and B are not identical. In that case, you can use a .csv file with four columns:

- lambda_A: The wavelengths recorded in state A.
- values_A: The intensity value of the sensor's output in state A.
- lambda_B: The wavelengths recorded in state B.
- values_B: The intensity value of the sensor's output in state B.

Once you have uploaded your spectra, you can use the `sensorOverlord::spectraMatrixFromValues` function to create a *Spectra* object:

```
spectra <- read.csv("my_spectra.csv")

# Option 1
spectra_object <- spectraMatrixFromValues(
  lambdas_minimum = spectra$lambda,
  values_minimum = spectra$values_A,
  lambdas_maximum = spectra$lambda,
  values_maximum = spectra$values_B
)

# Option 2
spectra_object <- spectraMatrixFromValues(
  lambdas_minimum = spectra$lambda_A,
  values_minimum = spectra$values_A,
  lambdas_maximum = spectra$lambda_B,
  values_maximum = spectra$values_B
)
```

You can use the `newSensorFromSpectra()` function to turn the *Spectra* object into a *Sensor* object at a certain ratiometric wavelength pair:

```
sensor_object <- newSensorFromSpectra(spectra_object,
  c(510, 520), c(455, 465))
```

That sensor object will now contain estimates for the three required parameters:

```
Ra <- sensor_object@Rmin
Rb <- sensor_object@Rmax
delta2 <- sensor_object@delta
```

(10.2.2.2) Option 2: use a provided spectrum

You can access all sensor information from the database with `sensorOverlord::getDb()$find()`, which accepts JSON search queries. For more information, see the associated vignette, located at http://apfeldlab.github.io/SensorOverlord/articles/accessing-database.html. For example:

```
deGFP1_information <- getDb()$find('{"sensor_name" : "deGFP1"}')
print(deGFP1_information$sensor_readout)
#> [[1]]
#> [1] "emission ratiometric"
print(deGFP1_information$sensor_midpoint)
#> [[1]]
#> [1] 8.02
print(deGFP1_information$lambda1_recommended)
#> [1] 515
print(deGFP1_information$lambda2_recommended)
#> [1] 460
```

The actual spectra information is in a list form, so you can use the `apply` and `unlist` functions to obtain the spectra:

```
library(data.table)
deGFP1_spectra_information <- data.table(
  apply(
    deGFP1_information[c("lambda_min", "values_min",
                         "lambda_max", "values_max")],
    2, function(x) unlist(x))
  )
```

Then, follow the same instructions as if you had provided the spectra yourself: create a *Spectra* object, create a *Sensor* object at a specific wavelength pair, and then extract the relevant parameters:

```
deGFP_spectra <- spectraMatrixFromValues(
        deGFP1_spectra_information$lambda_min,
        deGFP1_spectra_information$values_min,
        deGFP1_spectra_information$lambda_max,
        deGFP1_spectra_information$values_max
    )

sensor_object <- newSensorFromSpectra(deGFP_spectra,
                                       c(510, 520), c(455, 465))

Ra <- sensor_object@Rmin
Rb <- sensor_object@Rmax
delta2 <- sensor_object@delta

print(Ra)
#> [1] 0.3330232
print(Rb)
#> [1] 0.6447556
print(delta2)
#> [1] 0.9611258
```

(10.2.2.3) Option 3: provide parameters directly

If you conducted the appropriate microscopy experiments, you may have also obtained exact values of R_a, R_b, and $\delta_{\lambda 2}$.

If that is the case, you can use them to directly make a *Sensor* object:

```
R_reduced <- 0.6
R_oxidized <- 5.2
delta2 <- 0.171

sensor_object <- new(
            "Sensor",
            Rmax = R_reduced,
            Rmin = R_oxidized,
            delta = delta2
        )
```

(10.2.3) Estimating microscopy error

In order to use the SensorOverlord framework, you need to provide an estimate of the error of your *R* measurements in your system of interest. There are various ways to accomplish this, as we demonstrate in Supplementary Note 3.

In *C. elegans*, we found that our errors in *R* were relative—that is, $R_{obs} = R_{true} \times (1 + error)$—, were invariant over the range of all possible R values, and varied between experiments. (Supplementary Note 3).

SensorOverlord can accommodate any error model that you find in your system. For example:

```
# Relative error of 2.8%, or 0.028*R
error_relative <- function(x) x*0.028

# Absolute error of +/- 0.028
error_absolute <- function(x) 0.028

# Quadratic error
error_other <- function(x) 0.0001*x^2 + 0.028*x + 0.5
```

For more information about incorporating different error models, see the “Customizing Error Model” vignette. For now, this tutorial will assume that you are using a relative error.

(10.3) Running the SensorOverlord model

After obtaining the requisite parameters, you should have access to a *Sensor* object and an error model. For example:

```
sensor_object <- new(
            "Sensor",
            Rmax = R_reduced,
            Rmin = R_oxidized,
            delta = delta2
        )

my_error <- function(x) x*0.028
```

To run the SensorOverlord model, you’ll need to turn your generic *Sensor* object into a specific redox, pH, or ligand-binding sensor, using the sensor’s midpoint potential. For example:

```
redox_sensor_object <-
  new(
    "redoxSensor",
    sensor_object,
    e0 = -265
  )
```

(10.3.1) Generating a table of predicted errors

Use the `error_df()` method, defined on *redoxSensor*, *pHSensor*, and *ligandSensor* objects, to generate a table of the inaccuracy of the biosensor at different values of redox potential, pH, or p[*Ligand*]. For example:

```
redox_sensor_error <- error_df(
  redox_sensor_object,
  c(0.028),
  Emin = -300,
  Emax = -150
)

summary(redox_sensor_error)
#>        E              Error          Inaccuracy            Rmin
#>  Min.   :-300.0   Min.   : 0.7329   Length:15001       Length:15001
#>  1st Qu.:-262.5   1st Qu.: 1.1032   Class :character   Class :character
#>  Median :-225.0   Median : 3.4355   Mode  :character   Mode  :character
#>  Mean   :-225.0   Mean   :    Inf
#>  3rd Qu.:-187.5   3rd Qu.:31.3965
#>  Max.   :-150.0   Max.   :    Inf
#>      Rmax               Name
#>  Length:15001       Length:15001
#>  Class :character   Class :character
#>  Mode  :character   Mode  :character
#>
#>
#>

library(ggplot2)
ggplot(redox_sensor_error, aes(x = E, y = Error)) +
  geom_line() +
  ylim(c(0, 10))
```

For more information, see the “Sensor Methods” reference section in the online documentation.

(10.3.2) Computing suitable ranges

`sensorOverlord` can also compute the ranges of redox potential, pH, or ligand concentration that a biosensor can measure to different accuracy thresholds. For example:

```
# The redox potentials this sensor can measure, with an error in R of
# 2% or 3%, to an accuracy of 1mV or 2mV
accurate_ranges <- ranges_df(redox_sensor_object,
                                  inaccuracies = c(0.02, 0.03),
                                  thresholds = c(1, 2))

accurate_ranges
#>   Sensor_Name Minimum Maximum Inaccuracy error_thresh
#> 1 Sensor_0.02 -252.89 -205.11       0.02            1
#> 2 Sensor_0.02 -264.09 -200.00       0.02            2
#> 3 Sensor_0.03 -243.66 -214.85       0.03            1
#> 4 Sensor_0.03 -257.68 -200.44       0.03            2
```

(10.3.3) Plotting results

The `plotProperty()` method will plot *R* vs the property of interest. For redox biosensors in equilibrium with the glutathione couple, this will be *R* vs E_{GSH} (the half-cell reduction potential of the glutathione couple) [6].

```
plotProperty(redox_sensor_object)
```

The `rangePlot()` method will plot the range of values a biosensor can measure to a certain accuracy.

```
rangePlot(redox_sensor_object, ranges = accurate_ranges)
```

For more information on plotting methods, see the “Sensor Methods” section in the documentation reference.

(10.4) Using the SensorOverlord web application

The SensorOverlord web application can be accessed at http://www.sensoroverlord.org. It has been tested on Firefox version 79 and Chrome version 84.0.4147.105 on Windows 10 and Ubuntu 20.04.

The “Home” tab provides some background on the purpose of the application, as well as basic instructions for the application’s use.

The “Main Analysis” tab contains the main application functionality. To analyze a sensor, either (i) choose a sensor from the database in the dropdown menu under “Select a sensor”, (ii)

upload a .csv file containing spectra information by using the "Upload Spectra" tab near the bottom of the page, or (iii) input custom biophysical parameters by using the "Input Characteristics" tab, also near the bottom of the page.

If you are using spectra, you can also select dual-excitation or dual-emission wavelengths near the middle of the page, under the "What wavelengths are you using to make your measurements?" heading. In this section, you can also specify the size of the excitation or emission bands for each wavelength: for example, a "First Wavelength" of "410" and a "Band Size (1st Wavelength)" of "20" will consider all λ_1 between 400 nm and 420 nm.

After choosing a sensor, you will also need to input (i) the accuracy in your measurements of R, under the "Relative Microscopy Imprecision" header, and (ii) the accuracy or accuracies at which you wish to make measurements, separated by commas under the "Accuracy (comma-separated)" header. For example, to analyze a redox sensor at 2% microscopy precision to an accuracy of 0.5 *mV* and 1.0 *mV*, you would enter "0.02" and "0.5,1.0" into fields (i) and (ii), respectively.

When you are satisfied with your inputted parameters, press the "Run/Update Analysis" button to generate the two figures that display the suitable ranges and the phase diagram of selected sensor in the rightmost panel.

References

1. Meyer AJ, Dick TP. Fluorescent protein-based redox probes. *Antioxid Redox Signal*. 2010;13(5):621-50.
2. Elsliger MA, Wachter RM, Hanson GT, Kallio K, Remington SJ. Structural and spectral response of green fluorescent protein variants to changes in pH. *Biochemistry*. 1999;38(17):5296-301.
3. Hanson GT, McAnaney TB, Park ES, Rendell ME, Yarbrough DK, Chu S, Xi L, Boxer SG, Montrose MH, Remington SJ. Green fluorescent protein variants as ratiometric dual emission pH sensors. 1. Structural characterization and preliminary application. *Biochemistry*. 2002;41(52):15477-88.
4. Dooley CT, Dore TM, Hanson GT, Jackson WC, Remington SJ, Tsien RY. Imaging dynamic redox changes in mammalian cells with green fluorescent protein indicators. *J Biol Chem*. 2004;279(21):22284-93.
5. Hanson GT, Aggeler R, Oglesbee D, Cannon M, Capaldi RA, Tsien RY, Remington SJ. Investigating mitochondrial redox potential with redox-sensitive green fluorescent protein indicators. *J Biol Chem*. 2004;279(13):13044-53.
6. Romero-Aristizabal C, Marks DS, Fontana W, Apfeld J. Regulated spatial organization and sensitivity of cytosolic protein oxidation in Caenorhabditis elegans. *Nature communications*. 2014;5:5020.
7. Cannon MB, Remington SJ. Re-engineering redox-sensitive green fluorescent protein for improved response rate. *Protein Sci*. 2006;15(1):45-57.
8. Meyer AJ, Brach T, Marty L, Kreye S, Rouhier N, Jacquot JP, Hell R. Redox-sensitive GFP in Arabidopsis thaliana is a quantitative biosensor for the redox potential of the cellular glutathione redox buffer. *The Plant journal : for cell and molecular biology*. 2007;52(5):973-86.
9. Schwarzlander M, Fricker MD, Muller C, Marty L, Brach T, Novak J, Sweetlove LJ, Hell R, Meyer AJ. Confocal imaging of glutathione redox potential in living plant cells. *J Microsc*. 2008;231(2):299-316.
10. Drevon D, Fursa SR, Malcolm AL. Intercoder Reliability and Validity of WebPlotDigitizer in Extracting Graphed Data. *Behav Modif*. 2017;41(2):323-39.
11. Lohman JR, Remington SJ. Development of a family of redox-sensitive green fluorescent protein indicators for use in relatively oxidizing subcellular environments. *Biochemistry*. 2008;47(33):8678-88.
12. Cannon MB, Remington SJ. Redox-sensitive green fluorescent protein: probes for dynamic intracellular redox responses. A review. *Methods in molecular biology*. 2008;476:51-65.
13. Hanson GT. Green Fluorescent Protein Biosensors, PhD Thesis [PhD Thesis]: University of Oregon; 2001.
14. Aller I, Rouhier N, Meyer AJ. Development of roGFP2-derived redox probes for measurement of the glutathione redox potential in the cytosol of severely glutathione-deficient rml1 seedlings. *Front Plant Sci*. 2013;4:506.
15. Campbell BC, Petsko GA, Liu CF. Crystal Structure of Green Fluorescent Protein Clover and Design of Clover-Based Redox Sensors. *Structure*. 2018;26(2):225-37 e3.

16. Morgan B, Sobotta MC, Dick TP. Measuring E(GSH) and H2O2 with roGFP2-based redox probes. *Free Radic Biol Med*. 2011;51(11):1943-51.

17. Cannon MB. Re-engineering redox -sensitive green fluorescent protein as indicators of cellular thiol oxidation status, PhD Thesis [PhD Thesis]: University of Oregon; 2005.

18. Gutscher M, Pauleau AL, Marty L, Brach T, Wabnitz GH, Samstag Y, Meyer AJ, Dick TP. Real-time imaging of the intracellular glutathione redox potential. *Nat Methods*. 2008;5(6):553-9.

19. Barondeau DP, Putnam CD, Kassmann CJ, Tainer JA, Getzoff ED. Mechanism and energetics of green fluorescent protein chromophore synthesis revealed by trapped intermediate structures. *Proc Natl Acad Sci U S A*. 2003;100(21):12111-6.

20. Martynov VI, Pakhomov AA, Deyev IE, Petrenko AG. Genetically encoded fluorescent indicators for live cell pH imaging. *Biochim Biophys Acta Gen Subj*. 2018;1862(12):2924-39.

21. Baird GS, Zacharias DA, Tsien RY. Biochemistry, mutagenesis, and oligomerization of DsRed, a red fluorescent protein from coral. *Proc Natl Acad Sci U S A*. 2000;97(22):11984-9.

22. Arosio D, Ricci F, Marchetti L, Gualdani R, Albertazzi L, Beltram F. Simultaneous intracellular chloride and pH measurements using a GFP-based sensor. *Nat Methods*. 2010;7(7):516-8.

23. Arosio D, Garau G, Ricci F, Marchetti L, Bizzarri R, Nifosi R, Beltram F. Spectroscopic and structural study of proton and halide ion cooperative binding to gfp. *Biophys J*. 2007;93(1):232-44.

24. Bizzarri R, Arcangeli C, Arosio D, Ricci F, Faraci P, Cardarelli F, Beltram F. Development of a novel GFP-based ratiometric excitation and emission pH indicator for intracellular studies. *Biophys J*. 2006;90(9):3300-14.

25. Miesenbock G, De Angelis DA, Rothman JE. Visualizing secretion and synaptic transmission with pH-sensitive green fluorescent proteins. *Nature*. 1998;394(6689):192-5.

26. Mahon MJ. pHluorin2: an enhanced, ratiometric, pH-sensitive green florescent protein. *Adv Biosci Biotechnol*. 2011;2(3):132-7.

27. Rajendran M, Claywell B, Haynes EP, Scales U, Henning CK, Tantama M. Imaging pH Dynamics Simultaneously in Two Cellular Compartments Using a Ratiometric pH-Sensitive Mutant of mCherry. *ACS Omega*. 2018;3(8):9476-86.

28. Tantama M, Hung YP, Yellen G. Imaging intracellular pH in live cells with a genetically encoded red fluorescent protein sensor. *J Am Chem Soc*. 2011;133(26):10034-7.

29. Ermakova YG, Pak VV, Bogdanova YA, Kotlobay AA, Yampolsky IV, Shokhina AG, Panova AS, Marygin RA, Staroverov DB, Bilan DS, Sies H, Belousov VV. SypHer3s: a genetically encoded fluorescent ratiometric probe with enhanced brightness and an improved dynamic range. *Chem Commun (Camb)*. 2018;54(23):2898-901.

30. Matlashov ME, Bogdanova YA, Ermakova GV, Mishina NM, Ermakova YG, Nikitin ES, Balaban PM, Okabe S, Lukyanov S, Enikolopov G, Zaraisky AG, Belousov VV. Fluorescent ratiometric pH indicator SypHer2: Applications in neuroscience and regenerative biology. *Biochimica et biophysica acta*. 2015;1850(11):2318-28.

31. Poburko D, Santo-Domingo J, Demaurex N. Dynamic regulation of the mitochondrial proton gradient during cytosolic calcium elevations. *J Biol Chem*. 2011;286(13):11672-84.

32. Tao R, Zhao Y, Chu H, Wang A, Zhu J, Chen X, Zou Y, Shi M, Liu R, Su N, Du J, Zhou HM, Zhu L, Qian X, Liu H, Loscalzo J, Yang Y. Genetically encoded fluorescent sensors reveal dynamic regulation of NADPH metabolism. *Nat Methods*. 2017;14(7):720-8.

33. Shaner NC, Campbell RE, Steinbach PA, Giepmans BN, Palmer AE, Tsien RY. Improved monomeric red, orange and yellow fluorescent proteins derived from Discosoma sp. red fluorescent protein. *Nat Biotechnol*. 2004;22(12):1567-72.

34. Hu H, Gu Y, Xu L, Zou Y, Wang A, Tao R, Chen X, Zhao Y, Yang Y. A genetically encoded toolkit for tracking live-cell histidine dynamics in space and time. *Scientific reports*. 2017;7:43479.

35. Cambronne XA, Stewart ML, Kim D, Jones-Brunette AM, Morgan RK, Farrens DL, Cohen MS, Goodman RH. Biosensor reveals multiple sources for mitochondrial NAD(+). *Science*. 2016;352(6292):1474-7.

36. Zhao Y, Jin J, Hu Q, Zhou HM, Yi J, Yu Z, Xu L, Wang X, Yang Y, Loscalzo J. Genetically encoded fluorescent sensors for intracellular NADH detection. *Cell Metab*. 2011;14(4):555-66.
